# Characteristics of Spontaneous Anterior–Posterior Oscillation-Frequency Convergences in the Alpha Band

**DOI:** 10.1523/ENEURO.0033-24.2025

**Published:** 2025-03-25

**Authors:** Satoru Suzuki, Marcia Grabowecky, Melisa Menceloglu

**Affiliations:** ^1^Department of Psychology and Interdepartmental Neuroscience, Northwestern University, Evanston, Illinois 60208; ^2^Cognitive, Linguistic, and Psychological Sciences, Brown University, Providence, Rhode Island 02912

**Keywords:** alpha band, frequency convergence, human EEG, neural oscillation

## Abstract

Anterior–posterior interactions in the alpha band (8–12 Hz) have been implicated in various functions including perception, attention, and working memory. The underlying neural communication can be flexibly controlled by adjusting phase relations when activities across anterior–posterior regions oscillate at a matched frequency. We thus investigated how alpha oscillation frequencies spontaneously converged along anterior–posterior regions by tracking oscillatory EEG activity while participants rested. As more anterior–posterior regions (scalp sites) frequency-converged, the probability of additional regions joining the frequency convergence increased, and so did oscillatory synchronization (i.e., oscillatory power) at participating regions, suggesting that anterior–posterior frequency convergences are driven by inter-regional entrainment. Notably, frequency convergences were accompanied by two types of approximately linear phase gradients, one progressively phase lagged in the anterior direction, the posterior-to-anterior (P-A) gradient, and the other progressively phase lagged in the posterior direction, the anterior-to-posterior (A-P) gradient. These gradients implied traveling waves propagating in the feedforward and feedback directions, respectively. Interestingly, while in natural viewing frequency convergences were accompanied by both gradient types (occurring at different frequencies) regardless of anterior–posterior routes, when the eyes were closed, the P-A and A-P gradients spatially segregated, channeling feedforward flows of information primarily through the midline and feedback flows primarily through each hemisphere. Future research may investigate how eye closure organizes information flows in this way and how it influences hierarchical information processing. Future research may also investigate the functional roles of frequency-convergence contingent traveling waves in contrast to those generated by other mechanisms.

## Significance Statement

Anterior–posterior interactions in the alpha band (8–12 Hz) have been implicated in perception, attention, and working memory. While alpha frequencies differ across anterior–posterior regions, they also dynamically converge while people rest. Our EEG study investigated the mechanisms and functions of spontaneous alpha-frequency convergences. Our results suggest that anterior–posterior frequency convergences are driven by inter-regional entrainment. Notably, frequency convergences were accompanied by approximately linear posterior-to-anterior and anterior-to-posterior phase gradients, likely facilitating feedforward and feedback information flows via travelling waves. Interestingly, closing the eyes spatially organized these information flows, channeling feedforward flows through the midline and feedback flows through each hemisphere. Future research may investigate the behavioral significance of these frequency-convergence contingent flows of information.

## Introduction

Oscillatory neural activity is prevalent in the brain (Buzsaki, 2006). In particular, inter-regional phase locking of oscillatory activity has been proposed to play a crucial role in controlling neural communication (Fries, 2005, 2015), spurring much research examining inter-regional phase coherence within and between frequency bands. Most studies measured phase coherence as a period of stable phase difference, $\Delta \phi (t)=n\phi_{A}(t)-m\phi_{B}(t)\;∼\;\mathrm{constant}$, between signals from a pair of brain regions A and B at frequencies of a ratio, m:n [where $\phi_{A}(t)$ and $\phi_{B}(t)$ are instantaneous phases at regions A and B obtained by Hilbert transforming band-passed signals, convolving with Morlet wavelets, etc.]. For example, one would set m=n=1 for examining within-frequency phase coherence, m=1 and n=3 for examining cross-frequency phase coherence between f and 3×f, and so on. Those studies primarily focused on identifying frequency-specific functional networks organized by within- and/or cross-frequency phase coherence in specific frequency bands. Extensive research using this approach has identified a variety of frequency-specific functional networks and elucidated how they contribute to perceptual, attentional, and cognitive processes (Palva et al., 2005; Palva and Palva, 2012; Lobier et al., 2018; Marzetti et al., 2019).

Focusing on phase coherence in band-passed signals, however, obscures the fact that oscillation frequencies generally differ across brain regions (Keitel and Gross, 2016; Mahjoory et al., 2020) as well as fluctuate over time (Mierau et al., 2017; Benwell et al., 2019). In order for a pair of regions to stably interact through oscillatory phase locking, their oscillation frequencies need to converge (to the same frequency for within-frequency oscillatory interactions, which is the focus of the current study, or to frequencies of a ratio, m:n, for cross-frequency oscillatory interactions). Thus, a complementary approach to examining inter-regional oscillatory interactions is to track oscillation frequencies in multiple regions over time and examine how they dynamically converge. Oscillation frequency in each region (e.g., an intracranial electrode, EEG/MEG scalp site, inferred source, etc.) can be tracked by applying time–frequency decomposition (e.g., using Morlet wavelets) with appropriate spectral and temporal resolution, then tracking spectral-power ridges (or stationary-phase points where $\frac{d\mathrm{Phase}}{dt}=2\pi f$) as a function of time (Amor et al., 2005; Rudrauf et al., 2006; Mallat, 2009). Using this method, previous studies have examined spontaneous inter-regional oscillation–frequency convergences in preseizure neural activity (Amor et al., 2005; Rudrauf et al., 2006) as well as frequency convergences to perceived visual flicker during binocular rivalry using frequency-tagged stimuli (Rudrauf et al., 2006).

Our goal here was to uncover macroscopic rules governing spontaneous inter-regional oscillation–frequency convergences during rest using scalp-recorded EEG. What mechanisms drive oscillation frequencies in different regions to converge? What functions might inter-regional oscillation–frequency convergences serve? We sought clues to answering these questions by examining the spreading of oscillation-frequency convergences in relation to oscillatory power and phase relations.

We focused on oscillation-frequency convergences along the anterior–posterior axis partly because neural communication between anterior and posterior brain regions has been implicated in a variety of perceptual, attentional, and cognitive processes (de Pasquale et al., 2012; Gonzalez-Castillo and Bandettini, 2018; Lobier et al., 2018; Marzetti et al., 2019; Reinhart and Nguyen, 2019; Mashour et al., 2020) and partly because consistent anterior–posterior gradients of neuroanatomical and neurophysiological features have been reported, including neuron density, myelination, cortical thickness, functional-connectivity patterns, temporal integration, and oscillation frequency (Burt et al., 2018; Huntenburg et al., 2018; Mahjoory et al., 2020; Smith et al., 2024). We tracked oscillation frequencies in the alpha band because alpha oscillations have been implicated in a variety of perceptual, attentional, and memory processes (Palva and Palva, 2007; Busch and VanRullen, 2010; Mathewson et al., 2010; Klimesch, 2012; Bonnefond and Jensen, 2015; Fries, 2015; Harris et al., 2018; Lobier et al., 2018; Reinhart and Nguyen, 2019), have been shown to influence gamma band activity (Bahramisharif et al., 2013; Esghaei et al., 2022), and may organize information flow (Jensen et al., 2014; Muller et al., 2018; Zhang et al., 2018).

We examined the dynamics of spontaneous anterior–posterior oscillation-frequency convergences in an extended alpha range (5–15 Hz, accommodating the broad range, 6–13 Hz, of individual differences in primary alpha oscillation frequency; Chapeton et al., 2019) in representative rest conditions, while participants rested with their eyes closed, rested with their eyes open in a dark room, or viewed a silent nature video. We examined the spreading, power, and phase characteristics of inter-regional oscillation–frequency convergences to infer their potential mechanisms and functions. Overall, our results suggest that inter-regional entrainment drives anterior–posterior oscillation-frequency convergences in the alpha band, which may coordinate feedforward and feedback flows of information via traveling waves.

## Materials and Methods

### Participants

Fifty-two Northwestern University students (35 women, one nonbinary; mean age of 20.8 years, ranging from 18 to 29 years, standard deviation of 2.5 years) gave informed written consent to participate for monetary compensation ($10 per hour). All participants were right-handed, had normal hearing and normal or corrected-to-normal vision, and had no history of concussion. They were tested individually in a dimly lit or darkened room in the period between 5/11/2017 and 1/27/2020. The study protocol was approved by the Northwestern University Institutional Review Board. A group of 24 individuals participated in a rest-with-eyes-closed condition in which EEG was recorded for ∼5 min while participants rested with their eyes closed and *ad libitum* engaged in spontaneous thoughts. A second (nonoverlapping) group of 24 individuals participated in a replication of the rest-with-eyes-closed condition and subsequently participated in a rest-with-eyes-open-in-the-dark condition which was the same as the former except that the room was darkened and participants kept their eyes open while blinking naturally. A third group of 21 individuals (17 of whom previously participated in the rest-with-eyes-closed condition) participated in a view-silent-nature-video condition in which EEG was recorded for ∼5 min while they viewed a silent nature video. To evaluate test–retest reliability, the view-silent-nature-video condition was run twice (20–30 min apart), referred to as earlier viewing and later viewing. A generic nature video was presented on a 13 inch 2017 MacBook Pro equipped with a 2880 (horizontal)-by-1800 (vertical)-pixel-resolution LCD display with normal brightness and contrast settings, placed 100 cm in front of participants, subtending ∼16° (horizontal)-by-10° (vertical) of visual angle. Subsets of these data were previously analyzed for different purposes (Menceloglu et al., 2020a,b, 2021a,b, 2024).

### EEG recording and preprocessing

EEG was recorded from 64 scalp electrodes (although we used a 64-electrode montage, we excluded signals from noise-prone electrodes, Fpz, Iz, T9, and T10, from analyses) at a sampling rate of 512 Hz using a BioSemi ActiveTwo system (see www.biosemi.com for details), while participants rested with their eyes closed, rested with their eyes open in the dark, or viewed a silent nature video for ∼5 min. Electrooculographic activity was monitored using four face electrodes, one placed lateral to each eye and one placed beneath each eye. Two additional electrodes were placed on the left and right mastoids. The EEG data were preprocessed using EEGLAB and ERPLAB toolboxes for MATLAB (Delorme and Makeig, 2004; Lopez-Calderon and Luck, 2014). The data were rereferenced off-line to the average of the two mastoid electrodes and high-pass filtered at 0.01 Hz to remove drift.

### Estimating dura sources by surface-Laplacian transforming EEG signals

EEG source reconstruction methods constrained by structural MRI and fMRI localizers obtained from each participant can achieve superior source reconstruction with a brain model customized for each participant (Cottereau et al., 2015). Such an approach, however, was unavailable to us as we had neither structural MRI nor fMRI data for our participants. Among the noncustomized source-imaging methods, we chose the surface-Laplacian transform that (theoretically) estimates the spatial distribution of macroscopic current sources and sinks on the dura surface. The surface-Laplacian transform has been shown to produce similar sources to those inferred by deconvolving scalp EEG potentials using a generic model of thicknesses and impedances of the scalp and skull (Nunez et al., 1994). The surface-Laplacian transform has also been shown to approximate simulated sources and/or to extract neural correlates of some behavioral performances to a similar degree to commonly used source-imaging methods such as sLORETA and Beamforming (Tenke and Kayser, 2012; Cohen, 2015). Furthermore, there is no evidence (to our knowledge) to suggest that the latter source-imaging methods provide greater spatial resolution than the surface-Laplacian transform. Thus, our preference was to use the surface-Laplacian transform which is the most general source-imaging method that relies the least on model-specific assumptions and free parameters (Hjorth, 1980; Nunez et al., 1994; Kayser and Tenke, 2006; Nunez and Srinivasan, 2006; Tenke and Kayser, 2012).

The surface-Laplacian transform is expected to reduce volume conduction effects from substantially larger than 5 cm in raw EEG to within 1–3 cm (Nunez et al., 1994; Tenke and Kayser, 2012; Cohen, 2014; Menceloglu et al., 2024) which approximately corresponded to the average spacing of electrodes in our 64-channel montage. For our implementation of the surface-Laplacian transform, we used Perrin and colleagues’ algorithm (Perrin et al., 1987, 1989, 1990) with a “smoothness” value, *λ* = 10^−5^ (recommended for 64 channels; Cohen, 2014). We surface-Laplacian transformed all EEG signals and refer to those signals that represent the macroscopic current sources and sinks on the dura surface under the 60 scalp sites (with the four noise-prone sites excluded from the analyses) simply as EEG signals.

### EEG analysis

#### Taking the temporal derivative

We needed to track spectral ridges (likely indicative of oscillation frequencies) at subsecond temporal resolution. The aperiodic $1/f^{\beta }$ spectral background in EEG may interfere with the identification of spectral ridges. A commonly employed strategy to circumvent this problem is to compute FFTs over partially overlapping time windows of several seconds or longer and then fit a power function to each time-windowed FFT to remove the $1\;/f^{\beta }$ component (Chiang et al., 2011; van Albada and Robinson, 2013; Donoghue et al., 2020). However, it is difficult to reliably estimate the $1\;/f^{\beta }$ component on a subsecond timescale. Note that β in the $1\;/f^{\beta }$ spectral background varies around the value of 1 (see He, 2014, for a review of the various factors that influence β, and Gao et al., 2017, for contributions of excitatory and inhibitory dynamics to β). Although taking the temporal derivative of EEG ( $\frac{\Delta \mathrm{EEG}}{\Delta t}$, where Δt is the temporal resolution, i.e., 1/512 s) would completely neutralize the $1\;/f^{\beta }$ component only when β=1, the method worked well in our prior studies (Menceloglu et al., 2021a,b, 2024, Smith et al., 2024). Specifically, any time series, f(t), can be expressed as an integral over its sinusoidal components, $f(t)=\frac{1}{\sqrt{2\pi }}\int_{-\infty }^{\infty }g(\omega )e^{i\omega t}d\omega$ with $g(\omega )=\frac{1}{\sqrt{2\pi }}\int_{-\infty }^{\infty }\;f(t)e^{i\omega t}dt$, where ω/2π is frequency (Fourier's theorem). Because taking the temporal derivative merely multiplies each sinusoidal component by its frequency (from the chain rule of differentiation), taking the temporal derivative adds a log–log slope of 1 to an amplitude spectrum to cancel the log–log slope of the aperiodic component, which is −1 if β=1. If β is different than 1, taking the temporal derivative will either under- or overcompensate for the negative aperiodic slope. For the EEG data analyzed here, taking the temporal derivative effectively reduced or removed the negative slopes of the aperiodic components (not shown, but equivalent to the comparison plots shown for similar data in Fig. 1 in Smith et al., 2024).

#### Time–frequency decomposition using Morlet wavelets

To track EEG power spectra at high temporal and spectral resolutions, we used a Morlet wavelet-convolution method suitable for time–frequency decomposition of signals containing multiple oscillatory sources of different frequencies (see Cohen, 2014 for a review of different methods for time–frequency decomposition). Each Morlet wavelet is a Gaussian-windowed complex sinusoidal template characterized by its frequency as well as its temporal and spectral widths that limit its temporal and spectral resolution. We convolved each EEG waveform (i.e., its temporal derivative) with a set of wavelets tuned to a range of frequencies, yielding a time series of complex values per wavelet frequency. The power and phase of each extracted sinusoidal component at each timepoint were given by the modulus squared (power) and the arc tangent of the ratio of the imaginary component to the real component (phase). We used a set of wavelets with 160 frequencies, $f_{w}^{\prime }s$, ranging from 5 to 15 Hz (accommodating individual differences, 6–13 Hz, in primary alpha oscillation frequency; Chapeton et al., 2019). The $f_{w}^{\prime }s$ were logarithmically spaced as neural temporal-frequency tunings tend to be approximately logarithmically scaled (Hess and Snowden, 1992; Lui et al., 2007). The accompanying m parameter (roughly the number of cycles per wavelet, with the precise definition, m=2πf⋅SD, where SD is the wavelet standard deviation) was also logarithmically spaced between 11.7 and 35, yielding a temporal resolution of SD=370ms and a spectral resolution of FWHM(full-widthathalf-maximumofwaveletspectrum)=1.0Hz that were virtually invariant across the wavelet frequencies.

#### Identifying oscillation frequencies per timepoint

**Figure 1.**
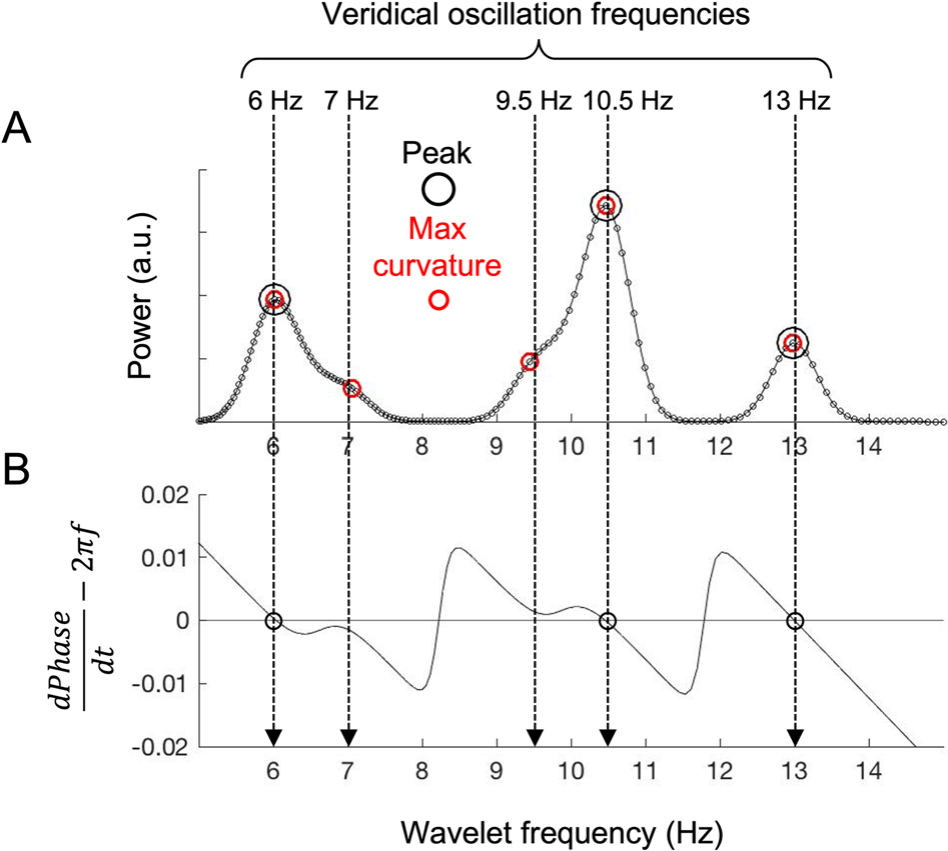
Identifying oscillation frequencies as curvature maxima in a power spectrum. ***A***, A power spectrum (for a single timepoint) of simulated EEG data containing sinusoidal oscillations at 6, 7, 9.5, 10.5, and 13 Hz (vertical arrows) of various amplitudes obtained with our 160 wavelets tuned to log-spaced frequencies between 5 and 15 Hz. The large black circles indicate oscillation frequencies identified as spectral peaks, whereas the red circles indicate oscillation frequencies identified as curvature maxima. Note that only curvature maxima identified the low-amplitude oscillations at 7 and 9.5 Hz occurring near the high-amplitude oscillations at 6 and 10.5 Hz. ***B***, A plot of $\frac{d\mathrm{Phase}}{dt}-2\pi f$ as a function of f (wavelet frequency). The negatively sloped zero crossings (black circles) indicate the points of phase stationarity, which coincide with the spectral peaks in ***A***.

Oscillation frequencies are typically identified as spectral peaks (a.k.a. ridges) that theoretically coincide with points of phase stationarity (Mallat, 2009). However, when multiple oscillations at neighboring frequencies coincide, each oscillation frequency does not necessarily generate a local maximum in the power spectrum due to limited spectral resolution. An example is shown in Figure 1*A*, plotting a power spectrum at a single timepoint obtained with our Morlet wavelets for simulated data containing five sinusoidal oscillations at 6, 7, 9.5, 10.5, and 13 Hz (vertical arrows) of different amplitudes. Only three of the five oscillation frequencies were identified as spectral peaks (Fig. 1*A*, black open circles) which coincided with points of phase stationarity (the negatively sloped zero crossings of $\frac{d\mathrm{Phase}}{dt}-2\pi f$ shown in Fig. 1*B*). Overshadowed by the neighboring high-amplitude oscillations at 6 and 10.5 Hz, the low-amplitude oscillations at 7 and 9.5 Hz did not generate peaks (Fig. 1*A*). However, it is evident that the low-amplitude oscillations generated “shoulders” within the larger profiles generated by the neighboring high-amplitude oscillations. Note that these spectral shoulders as well as spectral peaks generate curvature maxima. We thus identified oscillations as curvature maxima. As shown in Figure 1*A*, the low-amplitude oscillations at 7 and 9.5 Hz were successfully detected as curvature maxima (red open circles) though they generated neither spectral peaks nor points of phase stationarity.

The location of each spectral peak was identified as the frequency at which the power value was higher than those at the neighboring sampled frequencies. The spectral curvature at each frequency was computed discretely as the second derivative of spectral power (with respect to frequency), $\mathrm{Po}{w^{\prime \prime }}_{\;f_{i}}=(\mathrm{Po}w_{\;f_{i+1}}-\mathrm{Po}w_{\;f_{i}})-(\mathrm{Po}w_{\;f_{i}}-\mathrm{Po}w_{\;f_{i-1}})$, where $\mathrm{Po}{w^{\prime \prime }}_{\;f_{i}}$ is the second derivative at frequency $f_{i}$, $\mathrm{Po}w_{\;f_{i}}$ is the power value at $f_{i}$, and $\mathrm{Po}w_{\;f_{i+1}}$ and $\mathrm{Po}w_{\;f_{i-1}}$ are the power values at the neighboring sampled frequencies. The location of each curvature maximum was identified as the frequency at which $\mathrm{Po}w^{\prime \prime }$ was negative and more negative than those at the neighboring sampled frequencies (given that negative local minima of the second derivative correspond to spectral bumps).

We tracked curvature maxima as a function of time at anterior–posterior sites along the midline (AFz, Fz, FCz, Cz, CPz, Pz, POz, and Oz), left-hemisphere (AF3, F3, FC3, C3, CP3, P3, PO3, and O1), and right-hemisphere (AF4, F4, FC4, C4, CP4, P4, PO4, and O2) routes. It is likely that some of the curvature maxima associated with low powers were due to noise. Nevertheless, instead of imposing an arbitrary power threshold, we analyzed data relative to their time-shuffled controls as described below. The effects of spurious curvature maxima due to noise were discounted by comparing actual data with their time-shuffled controls.

#### Computing a frequency-convergence matrix containing frequency convergence sizes (one through eight sites) across frequencies (rows) and timepoints (columns)

We considered oscillation frequencies from a pair of sites to be converged when they were within ±1.4%. This was equivalent to having an overlap when each oscillation frequency was broadened by ±1 log unit (recall that we logarithmically sampled wavelet frequencies). This ±1.4% criterion balanced precision in detecting frequency convergences with the need for detecting sufficient instances of frequency convergences for meaningful statistical analysis.

For each site (for each participant and behavioral condition), we organized the time-varying oscillation frequencies in an oscillation-frequency-(row)-by-time-(column) matrix. An *oscillation-frequency matrix* was initially filled with zeros. Then, we filled the matrix element corresponding to each identified oscillation frequency at each timepoint as well as the rows immediately above and below it with ones (broadening each oscillation frequency by ±1 log unit; see above). Thus, each oscillation frequency at each timepoint was indicated by a vertical triplet of ones. To obtain a *frequency-convergence matrix* filled with oscillation-frequency convergence sizes (one through eight sites), we stacked the oscillation-frequency matrices from eight anterior–posterior sites on top of one another and summed them across the vertical dimension, yielding an oscillation-frequency-(row)-by-time-(column) matrix filled with values 1 through 8 corresponding to frequency-convergence sizes.

**Figure 2.**
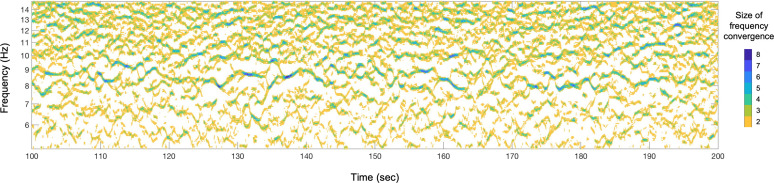
Visualization of a frequency-convergence matrix, showing the number of frequency-converged anterior–posterior sites (color coded) along the midline as a function of oscillation frequency (*y*-axis) and time (*x*-axis), for one participant in the rest-with-eyes-closed condition during a 100 s period. Cooler/darker colors indicate oscillation-frequency convergences across larger numbers of anterior–posterior sites.

How oscillation frequencies dynamically converged across anterior–posterior sites can be visualized by plotting a frequency-convergence matrix. An example is shown in Figure 2 for one participant in the rest-with-eyes-closed condition; the cooler/darker colors indicate frequency convergences (along the midline) across larger numbers of anterior–posterior sites.

#### Using time-shuffled frequency-convergence matrices as a control

Some of the observed oscillation-frequency convergences may be due to coincidences (across anterior–posterior sites) of alpha oscillations at dominant frequencies (∼10 Hz) and/or coincidences of low-power oscillations attributable to noise. To discount these spurious contributions, we generated time-shuffled frequency-convergence matrices. Specifically, we randomly time-shifted (in a circular manner) the oscillation-frequency matrix from each site (300 s long) by between 0 and 150 s (sampled from a uniform distribution). We then vertically summed the randomly time-shifted oscillation–frequency matrices across sites to generate a time-shuffled frequency-convergence matrix. To evaluate various characteristics of oscillation-frequency convergence, we computed the relevant measures with 20 time-shuffled frequency-convergence matrices and averaged them to generate their control values.

## Results

Our goal was to uncover general characteristics of spontaneous anterior–posterior oscillation-frequency convergences to infer their mechanisms and functions. To reveal any topographic characteristics, we examined anterior–posterior oscillation-frequency convergences along the midline route and the route through each hemisphere.

### Oscillation-frequency convergences are not mere coincidences

**Figure 3.**
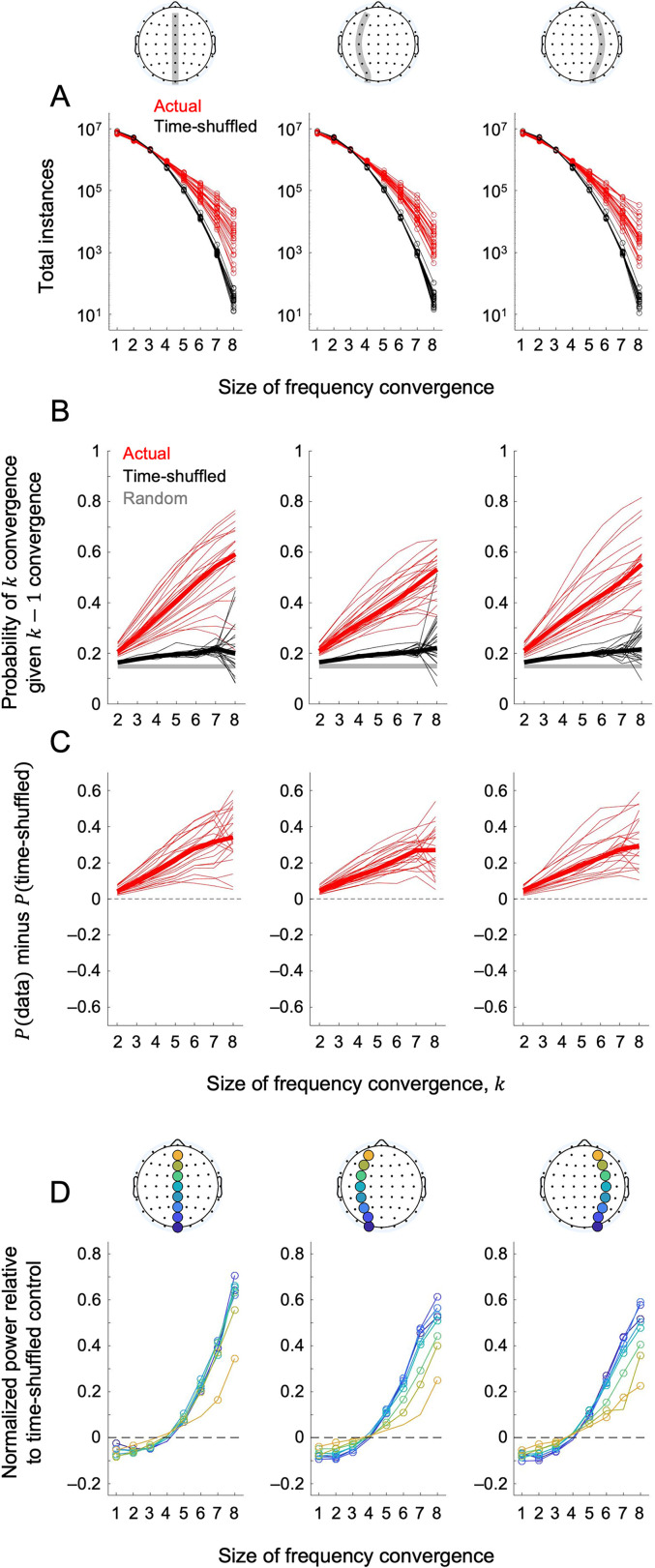
Instance, probability, and oscillatory power associated with oscillation-frequency convergence. Data for the rest-with-eyes-closed condition are presented as a representative example. ***A***, Total instances of anterior–posterior oscillation-frequency convergence as a function of the size of frequency convergence, for the midline (left), left-hemisphere (middle), and right-hemisphere (right) routes. The actual data are shown in red, and the time-shuffled controls are shown in black. Each curve represents a participant. Note that the instances of frequency convergence of four or more sites were elevated in the actual data (red) relative to the time-shuffled controls (black) for all participants. Data for all behavioral conditions are presented in Extended Data Figure 3-1. ***B***, The probability of k convergence (oscillation-frequency convergence across k sites) given k−1 convergence, that is, the probability of growing in convergence from k−1 to k, P(k|k–1), as a function of k, for the midline (left), left-hemisphere (middle), and right-hemisphere (right) routes. P(k|k–1) for the actual data (red) are shown with the corresponding time-shuffled controls (black) as well as P(k|k–1) expected if oscillation-frequency convergences occurred randomly (gray). ***C***, P(k|k–1) for the actual data minus that for the corresponding time-shuffled controls. The thin lines represent data from individual participants with the thick line representing the median. Note that the probability of incorporating another site into a frequency convergence increased as more sites frequency-converged. For (***B***) and (***C***), data for all behavioral conditions are presented in Extended Data Figure 3-2. ***D***, Time-averaged oscillatory power as a function of the size of frequency convergence at each site along the anterior–posterior axis [color-coded from dark blue (posterior) to yellow (anterior)], for the midline (left), left-hemisphere (middle), and right-hemisphere (right) routes. The power values are relative to those obtained with the corresponding time-shuffled controls, normalized to the maximum actual power for each power-by-convergence curve (per site per participant). The values averaged across participants are plotted, with each open circle indicating Bonferroni-corrected statistical significance (*α* = 0.05, two-tailed) relative to zero (control levels). Note that most sites showed accelerated increases in power when they participated in frequency convergences of four or more sites. Data for all behavioral conditions are presented in Extended Data Figure 3-3.

We first confirmed that anterior–posterior oscillation-frequency convergences occurred over and above what would be expected by random coincidences. We did so by comparing the total instances of frequency convergence of each size (one through eight) between the actual and time-shuffled frequency-convergence matrices. The instances of frequency convergence of four or more sites were consistently elevated in the actual data (red curves) relative to the time-shuffled controls (black curves) for all participants (Fig. 3*A* presenting the rest-with-eyes-closed condition as a representative example; see Extended Data Fig. 3-1 for the results from all behavioral conditions). Given that the total number of instances are plotted in a log scale, the elevations were substantial (by about an order of magnitude for convergence sizes of seven and eight). Thus, anterior–posterior oscillation-frequency convergences are coordinated rather than coincidental.

10.1523/ENEURO.0033-24.2025.f3-1 Figure 3-1 The same as Figure 3A, but shows data from all five behavioral conditions. Download Figure 3-1, TIF file.

10.1523/ENEURO.0033-24.2025.f3-2 Figure 3-2 The same as Figure 3B and 3C, but shows data from all five behavioral conditions. Download Figure 3-2, TIF file.

10.1523/ENEURO.0033-24.2025.f3-3 Figure 3-3 The same as Figure 3D, but shows data from all five behavioral conditions. Download Figure 3-3, TIF file.

To gain insights into the underlying mechanisms and functions of anterior–posterior oscillation-frequency convergences, we examined some of their general characteristics. While a variety of their spatial, spectral, and temporal features can be examined, we focused on the following questions. First, how does an oscillation-frequency convergence grow? For instance, does the probability of incorporating another site into a frequency convergence increase or decrease with the size of frequency convergence? Second, how are oscillation-frequency convergences related to oscillatory power? Third, are oscillation-frequency convergences accompanied by consistent phase relations? As discussed below, investigating these questions provided some insights into the potential mechanisms and functions of dynamic anterior–posterior oscillation-frequency convergences.

### The probability of incorporating another site into an oscillation-frequency convergence increased as the size of frequency convergence increased

What mechanisms drive anterior–posterior oscillation-frequency convergence? One possibility was that multiple regions could be driven to oscillate at a matched frequency via inter-regional entrainment. Spreading inter-regional entrainment would reciprocally enhance within-region synchronization (Tewarie et al., 2019), which in turn would facilitate further inter-regional spreading of entrainment. This possibility would predict that (1) a frequency convergence across more regions (sites) would be associated with higher oscillatory power (a measure of within-region synchronization) at participating sites and (2) the probability of another site joining a frequency convergence would increase as more sites frequency-converge. We evaluated Prediction 2 here and Prediction 1 in the next section.

We computed the probability of growing in frequency convergence as a function of the number of frequency-converged sites. We first obtained the average base probability of oscillatory activity by computing the proportion of ones in the oscillation-frequency matrix from each site (containing ones where oscillations were identified, broadened by ±1 log unit; see Materials and Methods) and averaging the values across sites. We call this P(1), the average probability of oscillatory activity. If oscillation-frequency convergences were due to random coincidences, the average probability of frequency convergence across k sites, P(k), should be given by $P(1{)}^{k}$.

We computed the probability of frequency convergences across k>1 sites as follows. For each combination of k sites, we stacked and summed their oscillation-frequency matrices and computed the proportion of k's in the resultant matrix to obtain the probability of k convergence. We then averaged the probabilities computed for all combinations of k sites to obtain the average probability of k convergence, P(k). To estimate the probability of growing in frequency convergence at each level of convergence, we computed the conditional probability of obtaining k convergence given k–1 convergence, P(k|k–1), by dividing P(k) by P(k–1) because k convergence was a subset of k–1 convergence. We computed P(k|k–1) (where 2≤k≤8) for each participant for each behavioral condition.

The probability of growing in convergence, P(k|k–1), monotonically increased as a function of the number of frequency-converged sites, k (red curves; Fig. 3*B* presenting the rest-with-eyes-closed condition as a representative example; see Extended Data Fig. 3-2*A* for the results from all behavioral conditions). Slight increases were also observed in the time-shuffled controls (black curves), indicating that some general features of the oscillation-frequency matrices (e.g., major oscillations occurring in relatively narrow frequency ranges) slightly increased the probability of coincidental frequency convergences relative to the chance level (gray line). The data for large frequency convergences (for k=7 and especially for k=8) were somewhat noisy as their instances were relatively rare especially for the time-shuffled controls (Fig. 3*A*; Extended Data Fig. 3-1). Importantly, P(k|k–1) for the actual data increased substantially more steeply than that for the time-shuffled controls as a function of k (Fig. 3*C* presenting the rest-with-eyes-closed condition as a representative example; see Extended Data Fig. 3-2*B* for the results from all behavioral conditions). This indicates that the probability of growing in frequency convergence monotonically increased as a function of the size of frequency convergence, controlling for any potential effects of time-independent spectral features at the individual sites.

### Larger oscillation-frequency convergences were associated with higher oscillatory power at participating sites

Here we evaluated the other prediction that if oscillation-frequency convergences were driven by inter-regional entrainment, frequency convergences across more regions (sites) would be associated with higher oscillatory power (a measure of within-region synchronization) at participating sites.

To evaluate this possibility, we temporally averaged the powers of all oscillations at each site, separately for each size of frequency convergence, k, in which it participated. For instance, we temporally averaged the powers of oscillations at AFz when their oscillation frequencies did not converge with any other sites (k=1), when their oscillation frequencies converged with one other site (k=2), their oscillation frequencies converged with two other sites (k=3), and so on. This yielded the average oscillatory power at each site as a function of the size of frequency convergence in which the site participated. However, we may obtain a spurious association between higher-power and larger-frequency convergence due to time-independent spectral features. For example, if higher-power oscillations concentrated within a narrow frequency range while lower-power oscillations broadly distributed across frequencies, higher-power oscillations would coincidentally frequency-converge with a higher probability than lower-power oscillations. We thus applied the same analysis to the corresponding time-shuffled controls (see Materials and Methods).

For each site, we computed its average oscillatory power minus the value obtained from the corresponding time-shuffled control as a function of the size of frequency convergence in which the site participated. To facilitate comparisons of these power-by-convergence curves across sites, participants, and behavioral conditions, we normalized each curve (actual minus control) by dividing it by its maximum actual power (per site per participant per behavioral condition).

Figure 3*D* shows the (normalized) average oscillatory power at each site [color coded from dark blue (posterior) to yellow (anterior)] as a function of the size of frequency convergence in which the site participated (the rest-with-eyes-closed condition presented as a representative example; see Extended Data Fig. 3-3 for the results from all behavioral conditions). The open circles indicate Bonferroni-corrected (for eight convergence sizes) significant difference from the control level (i.e., zero) at *α* = 0.05 (two-tailed). The oscillatory power generally increased as a function of the size of frequency convergence at all sites, rising significantly above the control levels at frequency convergences of four or five sites and steeply increasing at larger convergences (except that the power did not rise significantly above the control level at the anterior-most site during the natural viewing conditions; see Extended Data Fig. 3-3, yellow curves).

These results and those presented in the preceding section are consistent with the idea that anterior–posterior oscillation-frequency convergences are driven by inter-regional entrainment that reciprocally enhances within-region synchronization (this section), which in turn facilitates further inter-regional spreading of entrainment (preceding section).

### Anterior–posterior oscillation-frequency convergences were accompanied by approximately linear phase gradients

What functions might anterior–posterior oscillation-frequency convergences serve? When neural activities oscillate at a matched frequency across regions, communication between them can be controlled by adjusting phase lags. We thus examined how oscillation-frequency convergences influenced phase relations. For example, if frequency convergences were accompanied by linear phase gradients, one could infer that frequency convergences facilitate directional flows of information.

We computed sequential pairwise phase differences from posterior to anterior sites. Note that choosing this direction was arbitrary; the interpretation of the results would be the same regardless of the direction in which we computed phase differences. For example, for the midline route, we computed the phase of POz minus the phase of Oz, the phase of Pz minus the phase of POz, the phase of CPz minus the phase of Pz, the phase of Cz minus the phase of CPz, the phase of FCz minus the phase of Cz, the phase of Fz minus the phase of FCz, and the phase of AFz minus the phase of Fz. These sequential phase differences were computed at each oscillation frequency at each timepoint for each pair of sites that participated in a frequency convergence between two or more sites. Then, for each pair, their phase differences were averaged (as complex angles) across all instances, separately for each size of frequency convergence. Because of this temporal averaging, this analysis detected phase relations that were consistent over the ∼5 min period. The resultant time-averaged phase differences were averaged within each of the 19 partially overlapping frequency bins (1 Hz wide with 0.5 spacing).

**Figure 4.**
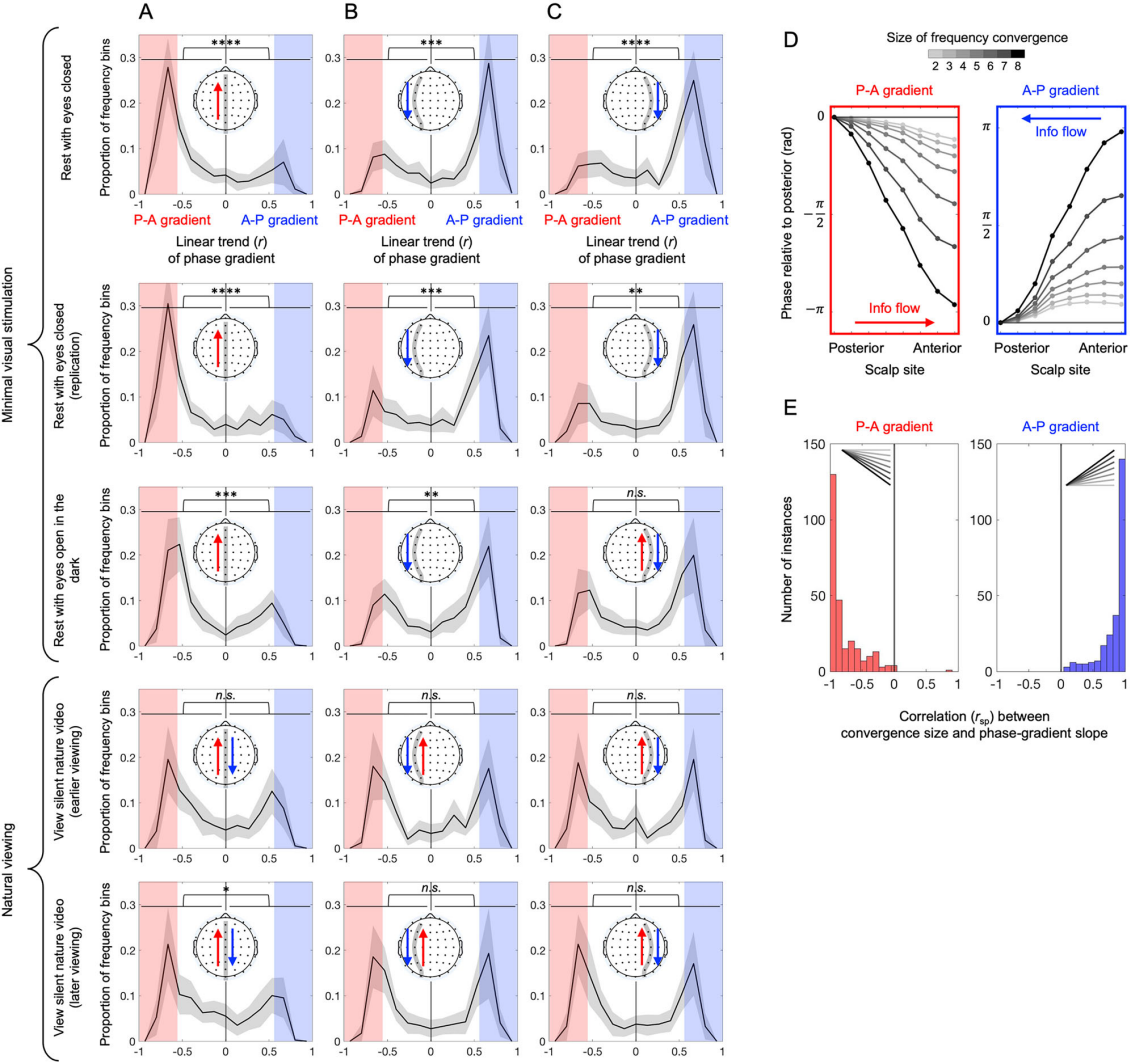
Characteristics of the anterior–posterior phase gradients accompanying oscillation-frequency convergence. ***A–C***, Distribution of the linear trend (Pearson's *r* weighted by PLV) of the anterior–posterior phase gradient (averaged across frequency-convergence sizes) obtained across the 19 frequency bins (1 Hz wide with 0.5 Hz spacing, spanning 5–15 Hz), for the midline (***A***), left-hemisphere (***B***), and right-hemisphere (***C***) routes. Negative values indicate the P-A gradients (with more anterior sites more phase lagged; illustrated with a single red arrow when they were prevalent), whereas positive values indicate the A-P gradients (with more posterior sites more phase lagged; illustrated with a single blue arrow when they were prevalent). The line represents the distribution averaged across participants, and the shaded area represents the 95% confidence interval. The rows represent the five behavioral conditions. Note that the distributions are bimodal for all anterior–posterior routes in all behavioral conditions, indicating that the P-A and A-P gradients are distinct types. The asterisks (*p < 0.05; **p < 0.01; ***p < 0.001; ****p < 0.0001) indicate *t* test results (two-tailed) comparing the number of negative with positive *r* values, testing the asymmetry of each distribution. Note that the distributions for the minimal-visual-stimulation conditions are mostly asymmetric (top three rows), whereas those for the natural viewing conditions are mostly symmetric (bottom two rows). ***D***, The P-A gradient (left; identified as the gradients with *r* less than −0.6; see the red-shaded regions in ***A–C***) and the A-P gradient (right; identified as the gradients with *r* greater than 0.6; see the blue-shaded regions in ***A–C***) averaged across the three anterior–posterior routes, participants, and behavioral conditions. Note that both gradients developed (i.e., became steeper) as more sites frequency-converged. ***E***, Histograms of the correlation (Spearman's *r*, *r*_sp_) between frequency-convergence size and phase-gradient slope for the P-A (left) and A-P (right) gradients (an entry per anterior–posterior route per participant per behavioral condition). As expected, the correlations were predominantly negative for the P-A gradients and predominantly positive for the A-P gradients, indicating that the phase gradients became steeper as more sites frequency-converged. Extended Data Figure 4-1 shows the correlation between the absolute slope of phase gradient (averaged across frequency-convergence sizes) and its average PLV across the 19 frequency bins. Extended Data Figure 4-2 shows the occurrences of the P-A and A-P gradients along each of the three anterior–posterior routes for each participant (per behavioral condition). Extended Data Figure 4-3 shows the distribution of the P-A and A-P gradients across the alpha range (5–15 Hz) along each of the three anterior–posterior routes (per behavioral condition). Extended Data Figure 4-4 shows the within-participant consistency of the alpha frequencies at which the P-A and A-P gradients formed during natural viewing (earlier vs later viewing of a silent nature video). Extended Data Figures 4-5–4-9 plot ***D*** and ***E*** separately for each of the three anterior–posterior routes and the five behavioral conditions. Extended Data Figure 4-10 plots ***D*** separately for three alpha subranges (5–8, 8–12, and 12–15 Hz). Extended Data Figure 4-11 is equivalent to ***D***, but the phase gradients were computed without surface-Laplacian transforming the EEG data.

To detect phase gradients (if any) in each frequency bin, we spatially integrated the corresponding sequential pairwise phase differences from the posterior to anterior sites (i.e., we cumulatively summed the sequential pairwise phase differences from the posterior to anterior sites) while arbitrarily setting the phase at the posterior-most site to zero. Overall, we observed two types of phase gradients (see below for details). One was negatively sloped (Fig. 4*D*, left) indicating that more anterior sites were more phase lagged, suggestive of a posterior-to-anterior flow of information, which we call the “posterior–anterior (P-A) gradient.” The other was positively sloped (Fig. 4*D*, right) indicating that more posterior sites were more phase lagged, suggestive of an anterior-to-posterior flow of information, which we call the “anterior–posterior (A-P) gradient.” It is clear from Figure 4*D* that both the P-A and A-P gradients developed, from barely sloped to substantially sloped, as the size of frequency convergence increased.

10.1523/ENEURO.0033-24.2025.f4-1 Figure 4-1 Histogram of the correlation (Pearson’s *r*) between the absolute linear slope of phase gradient (averaged across frequency-convergence sizes) and its average *PLV* (averaged across all sequential pairwise values within the gradient) across the 19 frequency bins. **A.** Histograms for the phase gradients along the midline route. **B & C.** Histograms for the phase gradients along the left-hemisphere and right-hemisphere routes. The rows represent the five behavioral conditions. Note that the average correlation was positive for all anterior-posterior routes and behavioral conditions, and statistically significant in most cases. This result indicates that steeper phase gradients tended to be temporally more stable. Download Figure 4-1, TIF file.

10.1523/ENEURO.0033-24.2025.f4-2 Figure 4-2 Linear trends of the anterior-posterior phase gradients (Pearson’s *r* weighted by *PLV*) across the 19 frequency bins (1 Hz wide with 0.5 Hz spacing) ordered from the lowest to highest *r* value for each participant (a line per participant). **A.** Phase gradients along the midline route. **B & C.** Phase gradients along the left- and right-hemisphere routes. Negative values indicate the P-A gradients (illustrated with a single red arrow when they were prevalent) whereas positive values indicate the A-P gradients (illustrated with a single blue arrow when they were prevalent). The histograms (of *r* values) on the right are the same as those shown in Figure 4A-4C. The rows represent the five behavioral conditions. Note that whether the P-A gradients dominated, A-P gradients dominated, or they formed equally (see the histograms and the red/blue arrows), both types of gradients formed at different alpha frequencies along each of the three anterior-posterior routes for most participants (i.e., most lines traverse high-negative and high-positive *r* values). Download Figure 4-2, TIF file.

10.1523/ENEURO.0033-24.2025.f4-3 Figure 4-3 Distribution of the linear trend of the anterior-posterior phase gradient (Pearson’s *r* weighted by *PLV*) obtained across the 19 frequency bins (1 Hz wide with 0.5 Hz spacing). **A.** Phase gradients along the midline route. **B & C.** Phase gradients along the left- and right-hemisphere routes. Negative values indicate the P-A gradients (illustrated with a single red arrow when they were prevalent) whereas positive values indicate the A-P gradients (illustrated with a single blue arrow when they were prevalent). The circles represent data from individual participants with the line representing the mean. The solid circles indicate Bonferroni-corrected statistical significance (*α* = 0.05, 2-tailed, relative to 0). The histograms (of *r* values) on the right are the same as those shown in Figure 4A-4C. The rows represent the five behavioral conditions. Note that the prevalent P-A gradients along the midline route (A) and the prevalent A-P gradients along the left- and right-hemisphere routes (B & C) in the eyes-closed conditions (top two rows) are broadly distributed over the alpha range. Download Figure 4-3, TIF file.

10.1523/ENEURO.0033-24.2025.f4-4 Figure 4-4 Histogram of the within-participant correlation (Pearson’s *r*) of the pattern of the negative and positive linear trends of the phase gradients across the 19 frequency bins between the earlier and later viewing of a silent nature video. **A.** The histogram for the midline route. **B & C.** The histograms for the left- and right-hemisphere routes. All distributions are significantly shifted to the right (the *p* values reflecting two-tailed *t*-tests), indicating that the alpha frequencies at which the P-A and A-P gradients formed along each of the three anterior-posterior routes tended to be consistent between the earlier and later viewing of a silent nature video for each participant. Download Figure 4-4, TIF file.

10.1523/ENEURO.0033-24.2025.f4-5 Figure 4-5 The same as Figure 4 but the plots here include only the rest-with-eyes-closed condition. The first row is the same as the first row in Figure 4A-4C. The second row is comparable to Figure 4D and the third row is comparable to Figure 4E. Download Figure 4-5, TIF file.

10.1523/ENEURO.0033-24.2025.f4-6 Figure 4-6 The same as Figure 4 but the plots here include only the rest-with-eyes-closed (replication) condition. The first row is the same as the second row in Figure 4A-4C. The second row is comparable to Figure 4D and the third row is comparable to Figure 4E. Download Figure 4-6, TIF file.

10.1523/ENEURO.0033-24.2025.f4-7 Figure 4-7 The same as Figure 4 but the plots here include only the rest-with-eyes-open-in-the-dark condition. The first row is the same as the third row in Figure 4A-4C. The second row is comparable to Figure 4D and the third row is comparable to Figure 4E. Download Figure 4-7, TIF file.

10.1523/ENEURO.0033-24.2025.f4-8 Figure 4-8 The same as Figure 4 but the plots here include only the view-silent-nature-video (earlier viewing) condition. The first row is the same as the fourth row in Figure 4A-4C. The second row is comparable to Figure 4D and the third row is comparable to Figure 4E. Download Figure 4-8, TIF file.

10.1523/ENEURO.0033-24.2025.f4-9 Figure 4-9 The same as Figure 4 but the plots here include only the view-silent-nature-video (later viewing) condition. The first row is the same as the fifth row in Figure 4A-4C. The second row is comparable to Figure 4D and the third row is comparable to Figure 4E. Download Figure 4-9, TIF file.

10.1523/ENEURO.0033-24.2025.f4-10 Figure 4-10 The same as Figure 4D, but the P-A and A-P gradients are plotted separately for three alpha subranges: 5-8 Hz, 8-12 Hz, and 12-15 Hz. Note that the gradients are generally similar across the alpha subranges. Download Figure 4-10, TIF file.

10.1523/ENEURO.0033-24.2025.f4-11 Figure 4-11 The same as Figure 4D, but the analysis was conducted without surface-Laplacian transforming the EEG data. Note that the phase-gradient slopes are decreased relative to Figure 4D by a factor of ∼10. Download Figure 4-11, TIF file.

To verify that these phase gradients accompanying oscillation-frequency convergences were distinctive types, we examined the distribution of the negative and positive phase gradients across the frequency bins. For each frequency bin, we computed the linear trend of the overall phase gradient (averaged across frequency-convergence sizes) by computing Pearson's *r* between the serial site index (1 through 8 from posterior to anterior) and the corresponding phase value. We weighted each *r* value by a measure of the temporal stability of the corresponding phase gradient, that is, by its average phase-locking value (PLV; Cohen, 2014; averaged across all sequential pairwise values within the gradient). We note that steeper phase gradients tended to have higher PLVs (especially in the minimal-visual-stimulation condition; Extended Data Fig. 4-1) indicating that steeper phase gradients tended to be temporally more stable. PLV's were corrected for sample-size–dependent biases by subtracting the expected chance levels (computed with the assumption of random sampling from a uniform angular distribution, well approximated by a power function of the sample size). As the corrected PLV's varied between close to 1, indicative of perfect phase locking, and near 0, indicative of no phase locking, the PLV-weighted *r* values varied between close to −1, indicative of a perfectly negative linear P-A gradient, to close to 1, indicative of a perfectly positive linear A-P gradient. For brevity, we will refer to the PLV-weighted *r* simply as *r*.

We made a histogram of the 19 *r* values (computed for the 19 frequency bins) for each participant. We then averaged the histograms across participants separately for the three anterior–posterior routes and the five behavioral conditions. The distributions were consistently bimodal (Fig. 4*A–C*), indicating that oscillation-frequency convergences were accompanied by two distinct types of phase gradients, the P-A gradients (with negative *r* values) and the A-P gradients (with positive *r* values).

Notably, in the minimal-visual-stimulation conditions (especially in the eyes-closed conditions), the P-A and A-P gradients were spatially segregated. Through the midline, oscillation-frequency convergences were predominantly accompanied by the P-A gradients, indicated by the dominant peak at a negative *r* value (Fig. 4*A*, top three rows). In contrast, through the left- and right-hemisphere, frequency convergences were predominantly accompanied by the A-P gradients, indicated by the dominant peak at a positive *r* value (Fig. 4*B,C*, top three rows). This spatial segregation disappeared during natural viewing. Frequency convergences were accompanied by about equal numbers of P-A and A-P gradients through the midline and each hemisphere, indicated by the symmetrically bimodal distributions of the *r* values (Fig. 4*A–C*, bottom two rows).

For nearly all participants for all anterior–posterior routes and behavioral conditions, oscillation-frequency convergences were accompanied by both the P-A and A-P gradients forming at different frequencies (Extended Data Fig. 4-2). The frequencies at which the two gradient types formed were not consistent across participants except that in the eyes-closed conditions either gradient type dominated (per anterior–posterior route) across a broad range of frequencies for most participants (Extended Data Fig. 4-3). Nevertheless, there was some within-participant consistency in the frequencies at which the two gradient types formed as the pattern of the negative and positive *r* values across frequency bins was significantly correlated between the earlier and later viewing of a silent nature video (Extended Data Fig. 4-4).

We next examined how the P-A and A-P gradients changed as a function of the size of frequency convergence. To consider robust cases of the P-A and A-P gradients (which occurred in specific frequency bins; Extended Data Figs. 4-2, 4-3), we selected the gradients with *r* values less than −0.6 (occupying mostly below the lower bimodal peak; Fig. 4*A–C*, highlighted with red-shaded regions) as representative P-A gradients and those with *r* values greater than 0.6 (occupying mostly above the upper bimodal peak; Fig. 4*A–C*, highlighted with blue-shaded regions) as representative A-P gradients. We then separately averaged these samples of the P-A and A-P gradients (taken from frequency bins in which they were robust) across the three anterior–posterior routes, participants, and behavioral conditions. As noted above, it is clear that both the P-A and A-P gradients developed, that is, changed from barely sloped to substantially sloped, as more sites frequency-converged (Fig. 4*D*). To confirm this observation, we computed the correlation (Spearman's *r*, *r*_sp_) between frequency-convergence size and phase-gradient slope (the slope of the linear fit going through the origin) separately for the P-A and A-P gradients per anterior–posterior route per participant per behavioral condition. For the P-A gradients, the *r*_sp_ values were predominantly negative (Fig. 4*E*, left) indicating that the negative slopes increased with the increasing frequency-convergence size. For the A-P gradients, the *r*_sp_ values were predominantly positive (Fig. 4*E*, right) indicating that the positive slopes increased with the increasing frequency-convergence size. This confirms that both the P-A and A-P gradients became steeper, that is, both phase gradients developed, as more sites frequency-converged. We further note that the phase gradients were approximately linear when all anterior–posterior sites frequency-converged (Fig. 4*D*, the darkest line), implying traveling waves propagating in the anterior and posterior directions (see Discussion below). Similar patterns of results were obtained separately in each behavioral condition (Extended Data Figs. 4-5–4-9).

### Are the observed oscillation-frequency convergences an artifact of source mixing (primarily volume conduction) effects?

In scalp-recorded EEG, source mixing (primarily volume conduction) effects are extensive (extending >5 cm; Schaworonkow and Nikulin, 2022). We used the surface-Laplacian transform to reduce source mixing effects to 1–3 cm (Nunez et al., 1994; Tenke and Kayser, 2012; Cohen, 2014). For the current dataset, we verified that volume conduction effects attenuated within ∼1.2 times the average interelectrode distance (Menceloglu et al., 2024; Fig. 4). Thus, the observed frequency convergences across four or more sites are unlikely to have been generated by volume conduction effects from a single oscillatory source. Importantly, a theoretical consideration and the characteristics of the phase gradients reasonably rule out the possibility that the observed oscillation-frequency convergences could have been spuriously generated by source mixing effects.

Given that source mixing effects are virtually instantaneous, any volume conducted oscillatory activity from a source detected elsewhere has a phase lag of zero. The phase gradients we observed therefore could not have been generated by source mixing effects. If anything, given the likely presence of at least some source mixing effects (especially between adjacent sites), the actual anterior–posterior phase gradients would have been steeper than what we observed. The reason is that any source mixing effects would reduce observed phase differences. Suppose oscillation at site2 was ahead of oscillation at site1 by Δθ, that is, site2=A2sin(2πft+Δθ) while site1=A1sin(2πft), where A2 and A1 are oscillation amplitudes at site2 and site1, f is the oscillation frequency, and t is time. Adding the volume conducted oscillation from site1, $A1_{\mathrm{VC}}\sin\;(2\pi ft)$, to oscillation at site2, A2sin(2πft+Δθ), yields $A^{\prime }\sin\;(2\pi ft+\Delta \theta^{\prime })$, where:

$$\Delta \;\theta^{\prime }=\mathrm{atan}[A2\sin\;(\Delta \theta )/\;(A1_{\mathrm{VC}}+A2\cos\;(\Delta \theta ))].$$

Given that $\Delta \theta^{\prime }<\Delta \theta$ (as $A1_{\mathrm{VC}}$ is in the denominator), source mixing effects would always reduce observed phase differences relative to the actual phase differences. Indeed, the slopes of the P-A and A-P gradients decrease by a factor of ∼10 if we do not reduce source mixing effects by applying the surface-Laplacian transform (compare Fig. 4*D* with Extended Data Fig. 4-11).

Furthermore, if oscillation-frequency convergences were driven by source mixing effects from a prominent source, frequency convergencies across more sites would be driven by more extensive source mixing effects from stronger oscillation at the source. Stronger source mixing effects (e.g., larger $A1_{\mathrm{VC}}$ in the above example) would have made the phase gradients less steep (see Eq. 1, having $A1_{\mathrm{VC}}$ in the denominator). To the contrary, we observed that the phase gradients became steeper as more sites frequency-converged (Fig. 4*D*,*E*).

## Discussion

Neural communication can be controlled by adjusting phase relations when activities in multiple regions oscillate at a matched frequency. We investigated how oscillation frequencies dynamically converged across regions. We focused on oscillation-frequency convergences along the anterior–posterior axis partly because anatomical, neurophysiological, and functional features exhibit systematic gradients along the anterior–posterior axis (Burt et al., 2018; Huntenburg et al., 2018; Mahjoory et al., 2020) and partly because anterior–posterior neural interactions have been implicated in a variety of perceptual, attentional, and memory processes (de Pasquale et al., 2012; Gonzalez-Castillo and Bandettini, 2018; Lobier et al., 2018; Marzetti et al., 2019; Reinhart and Nguyen, 2019; Mashour et al., 2020). We focused on an extended alpha range (5–15 Hz) because theta, alpha, and low-beta band oscillations are thought to be involved in organizing gamma-oscillation activity (Bahramisharif et al., 2013; Esghaei et al., 2022), controlling information flows (Jensen et al., 2014; Muller et al., 2018; Zhang et al., 2018), and mediating perceptual, attentional, and memory processes (Palva and Palva, 2007; Busch and VanRullen, 2010; Mathewson et al., 2010; Klimesch, 2012; Bonnefond and Jensen, 2015; Fries, 2015; Harris et al., 2018; Lobier et al., 2018; Reinhart and Nguyen, 2019). As we were interested in spontaneous oscillation-frequency convergences, participants rested with their eyes open or closed. To reveal any topographic characteristics, we examined oscillation-frequency convergences along three anterior–posterior routes, through the midline and each hemisphere.

What mechanisms drive anterior–posterior oscillation-frequency convergences? We showed that the probability of an additional site joining a frequency convergence monotonically increased as the size of frequency convergence increased, suggesting that entrainment of additional regions facilitates its spreading. We further showed that oscillatory power at each site increased as it participated in a more extensive frequency convergence. These results are consistent with the idea that anterior–posterior oscillation-frequency convergences are driven by inter-regional entrainment that reciprocally enhances within-region synchronization, which in turn facilitates further inter-regional spreading of entrainment. Future research may investigate what mechanisms trigger the process of inter-regional entrainment that leads to extensive anterior–posterior frequency convergence.

Notably, oscillation-frequency convergences were accompanied by two distinct phase gradients: the P-A gradients (with more anterior sites more phase lagged) and the A-P gradients (with more posterior sites more phase lagged). These gradients were minimal when only two sites frequency-converged but developed into robust approximately linear gradients when all anterior–posterior sites frequency-converged. This coemergence of frequency convergence and phase gradient suggests that they are generated by common mechanisms, which may be investigated in future research.

Regarding the potential functions of the frequency-convergence contingent phase gradients, the P-A gradients may facilitate information flows from posterior to anterior regions in the sense that oscillations ahead in phase could be driving those behind in phase. Similarly, the A-P gradients may facilitate information flows from anterior to posterior regions. Given that the phase gradients (on average) were approximately linear when all anterior–posterior sites frequency-converged and given that the scalp sites along the three anterior–posterior routes (the midline, left-hemisphere, and right-hemisphere routes) were approximately evenly spaced, the frequency-convergence contingent phase gradients imply traveling waves. The phase gradients accompanying full anterior–posterior frequency convergences traversed approximately ±π radians from the posterior-most to anterior-most site (Fig. 4*D*) in different subranges of the alpha band (Extended Data Fig. 4-10, darkest curves). Given that the anterior–posterior routes spanned ∼0.24 m along the scalp, the speed of the putative traveling waves ranged from ∼2.4 m/s at 5 Hz to ∼7.2 m/s at 15 Hz. This falls within the range (1–10 m/s) previously reported in macroscopic electrophysiological (i.e., EEG/MEG) recordings (Patten et al., 2012; Muller et al., 2018). The fact that frequency convergences were accompanied by both the P-A and A-P gradients at different alpha frequencies along each of the three anterior–posterior routes suggests that alpha band traveling waves propagate in the posterior-to-anterior (feedforward) and anterior-to-posterior (feedback) directions at different alpha frequencies through the midline as well as through the left and right hemispheres.

During natural viewing, the frequency-convergence contingent P-A and A-P gradients formed at different alpha frequencies (specific to individuals) evenly along each of the three anterior–posterior routes. Interestingly, under minimal visual stimulation, especially when the eyes were closed, the P-A gradients predominantly formed along the midline route, whereas the A-P gradients predominantly formed along the left- and right-hemisphere routes. This suggests that, whereas during natural viewing frequency convergences facilitate both feedforward and feedback flows of alpha band traveling waves through each of the three anterior–posterior routes, when the eyes are closed, feedforward flows are predominantly channeled through the midline while feedback flows are predominantly channeled through each hemisphere. Future research may investigate how eye closure organizes information flow in this way and how it influences hierarchical information processing.

Prior studies examining traveling waves in the alpha and other frequency bands in EEG reported that they tended to propagate from posterior to anterior regions when the eyes were open and from anterior to posterior regions when the eyes were closed (Alexander et al., 2013; Alamia and VanRullen, 2019). We did not find this asymmetry, but there is an important methodological difference between those studies and the current study. The prior studies identified traveling waves by detecting periods of spatiotemporally linear phase relations. They would have detected traveling waves when the oscillation frequencies were converged along their traveling paths (as in the current study) such as (1) when a single oscillatory population excited more distant neural populations with greater delays and/or (2) when an oscillatory excitation spread by sequentially (with a constant delay) activating a chain of neural populations. However, they would have also detected traveling waves when oscillation frequencies were not converged along their traveling path such as (3) when a chain of oscillatory neural populations influenced one another to generate a spatial gradient of oscillation frequencies wherein a wave travels from higher-frequency populations to lower-frequency populations (Ermentrout and Kleinfeld, 2001). It is possible that a large proportion of traveling waves detected in EEG recordings are generated by Mechanism 3, which would not be accompanied by frequency convergence. We suspect that those traveling waves dominated the results of the prior studies. In contrast, we selectively detected traveling waves accompanying oscillation-frequency convergences [e.g., those generated by Mechanism(s) 1 and/or 2 above]. Future research may investigate the functional roles of frequency-convergence contingent traveling waves in contrast to those generated by other mechanisms.

In summary, alpha oscillation frequencies spontaneously converge along the anterior–posterior axis while people rest, likely driven by inter-regional entrainment. Frequency convergences were accompanied by opposing phase gradients reflective of feedforward and feedback traveling waves which became spatially segregated when the eyes were closed. Future research may investigate the mechanisms that (1) trigger the inter-regional entrainment leading to large frequency convergences, (2) generate the associated phase gradients in specific directions, and (3) spatially segregate the opposing phase gradients upon eye closure, as well as the functional roles of frequency-convergence contingent traveling waves.

## Synthesis

Reviewing Editor: Anne Keitel, University of Dundee Decisions are customarily a result of the Reviewing Editor and the peer reviewers coming together and discussing their recommendations until a consensus is reached. When revisions are invited, a fact-based synthesis statement explaining their decision and outlining what is needed to prepare a revision will be listed below. The following reviewer(s) agreed to reveal their identity: Domenica Veniero, Felix Siebenhuhner.

Both original reviewers have assessed the revised manuscript which was then discussed with the editor. We would like to commend the authors for their thorough revision. Nonetheless, the reviewers had some remaining requests for further clarifications that should be addressed in a revised version. We therefore reached the decision "Revise and Resubmit".

Please find the unabridged reviewer comments below. These should be addressed in a point-by-point manner.

*****

Reviewer #1

Advances the Field (Required)

The authors report a straightforward method to investigate alpha convergence across sites, in different states. This is a relatively new way of looking at how information flows both in feedforward and feedback interactions. Methods and results might open new avenues for investigating how oscillations contribute to inter-areas communication.

Comments to the Authors (Required)

The authors have extensively revised the manuscript. the new version reads much better and highlights the importance of the work.

They have addressed all my comments satisfactorily. I only have 1 last comment. Will data and code be made available?

*****

Reviewer #2

Advances the Field (Required)

The manuscript introduces a novel approach to investigating frequency convergences which when combined with other analyses could provide new insights into the mechanisms of oscillations and synchronization.

Comments to the Authors (Required)

I find that the authors have taken considerable effort in addressing my comments and improving the manuscript; it is now much more readable. Also, the re-design and re-organization of figures has been well done. I do however still have a few comments and suggestions that should be addressed before the manuscript can be considered ready for publication.

Abstract:

Overall, the abstract is improved and easier to read. However, the abstract consists mostly of results, and very little background and not a lot of discussion. Maybe 1-2 more sentences at the beginning about what motivated this study could be added and some details of results dropped. For example, I would say that now there is too much use of "P-A" and "A-P" and "feedforward" and "feedback" in the abstract.

The sentence on lines 23-26 is difficult to understand, and should perhaps be summarized in a more succinct form, or left out.

The term "synergy" is used here without explanation of what it signifies - in fact, I have not found any explanation for it throughout the manuscript, contrary to what is claimed in the rebuttal. Maybe in the abstract and summary, it could be left out; and if it is used in the main text, its intended meaning should be explained in the introduction already.

Introduction:

The precise meaning of the term "synergistic" within this manuscript should be explained here, if the authors want to keep using that term.

Methods:

The description of how oscillation frequencies were identified is still lacking clarity. Most importantly, how were peaks and curvature maxima identified in practice in the analyses of the dataset? Visually or programmatically (i.e., with code)?

Also, "Curvature" needs to be explicitly defined mathematically.

The estimation of the "linear trend of the phase gradient" (l. 452) needs to be described in the methods.

Results:

l. 303 ff: This is unclear. Why are instances equal to durations? It is also not immediately intuitive why and how durations were overestimated.

l. 314 ff. This is written very vaguely: "elevations were substantial". Also, "some underlying mechanisms" - this is both vague and not well motivated.

l. 449 ff: The method for computing the "linear trend of the phase gradient" needs to be described in detail in the methods section. In the results section, there should be a shorter (yet understandable) description. With the current description, it is not very clear at all what was done here. The linear trend was estimated as the Pearson's r of which two variables? Over which dimension? (e.g. sites? frequencies? times points?) Why was the phase gradient weighted by its consistency? Why was PLV taken as a value of consistency? Was the tendency of PLV to report false positives from linear mixing accounted for mathematically?

l. 513ff. While this paragraph nicely addresses the question of spurious observations, there are some issues with it:

Volume conduction is only part of the potential sources of linear mixing (source spread being another), although a very large one in EEG. So maybe the section should be titled differently.

At least the equation 1 should be in display, not inline, mode

Maybe the equations should be moved to Methods section?

The results of the control analysis in which slopes were found to be less steep are an important finding and should be shown as extended data. I also recommend that this paragraph be re-written around the control analysis, so that it read less like a part of the Discussion

## Author Response

Response to each of the reviewers' comments. The reviewers' comments are italicized and our responses are in purple text.

Reviewer #1 Advances the Field (Required) The authors report a straightforward method to investigate alpha convergence across sites, in different states. This is a relatively new way of looking at how information flows both in feedforward and feedback interactions. Methods and results might open new avenues for investigating how oscillations contribute to inter-areas communication.

Comments to the Authors (Required) The authors have extensively revised the manuscript. the new version reads much better and highlights the importance of the work.

They have addressed all my comments satisfactorily. I only have 1 last comment. Will data and code be made available? We thank the reviewer for evaluating the revision. The raw and surface-Laplacian transformed EEG data are now available at https://doi.org/10.21985/n2-tfnq-tq65. The Matlab code we used to convolve EEG data with wavelets were adapted from Cohen's book (2014) (cited in the paper). For the more specific analyses described in the paper, we believe a reader should be able to write their own preferred code based on the descriptions we provide in the paper. Nevertheless, if anyone wants to test the reproducibility of our exact analyses and/or needs assistance with the presented analyses, we are happy to share and explain the Matlab code we used (though our code is neither the most readily decipherable nor the most efficient). ***** Reviewer #2 Advances the Field (Required) The manuscript introduces a novel approach to investigating frequency convergences which when combined with other analyses could provide new insights into the mechanisms of oscillations and synchronization.

Comments to the Authors (Required) I find that the authors have taken considerable effort in addressing my comments and improving the manuscript; it is now much more readable. Also, the re-design and re-organization of figures has been well done. I do however still have a few comments and suggestions that should be addressed before the manuscript can be considered ready for publication.

Abstract:

Overall, the abstract is improved and easier to read. However, the abstract consists mostly of results, and very little background and not a lot of discussion. Maybe 1-2 more sentences at the beginning about what motivated this study could be added and some details of results dropped. For example, I would say that now there is too much use of "P-A" and "A-P" and "feedforward" and "feedback" in the abstract.

The sentence on lines 23-26 is difficult to understand, and should perhaps be summarized in a more succinct form, or left out.

The term "synergy" is used here without explanation of what it signifies - in fact, I have not found any explanation for it throughout the manuscript, contrary to what is claimed in the rebuttal. Maybe in the abstract and summary, it could be left out; and if it is used in the main text, its intended meaning should be explained in the introduction already.

We appreciate these detailed comments as abstract is a prominent component of a research article. We have revised it as follows. (1) We have added a couple of introductory sentences while tightening the description of the results. (2) We have revised the lines 23-26 (regarding the spatial segregation of the P-A and A-P gradients) to improve clarity. (3) We originally used the term "synergy" to convey that the interactions propelling frequency convergences strengthened as more regions frequency converged, but we agree that "synergy" would not be an appropriate term for this. We now suggest that the observed oscillation-frequency convergences are driven by inter-regional entrainment. We explain that this interpretation is consistent with our finding that larger frequency convergences were associated with both stronger spreading of frequency convergences and increased oscillatory power at participating sites. We have removed the term "synergy" throughout the manuscript.

Introduction:

The precise meaning of the term "synergistic" within this manuscript should be explained here, if the authors want to keep using that term.

As indicated above, we have removed "synergy" and its derivatives throughout the manuscript.

Methods:

The description of how oscillation frequencies were identified is still lacking clarity. Most importantly, how were peaks and curvature maxima identified in practice in the analyses of the dataset? Visually or programmatically (i.e., with code)? Also, "Curvature" needs to be explicitly defined mathematically.

We apologize for this omission. We now describe the algorithm we used to compute powerspectrum peaks, curvature, and curvature maxima.

The estimation of the "linear trend of the phase gradient" (l. 452) needs to be described in the methods.

Please see our response to I. 449 ff below.

Results: l. 303 ff: This is unclear. Why are instances equal to durations? It is also not immediately intuitive why and how durations were overestimated.

We agree that the instance-to-duration conversion was confusing, and in fact it was unnecessary. To demonstrate that frequency convergences of larger sizes could not be due to coincidences, we just needed to show that the instances of large-size frequency convergences were substantially elevated in the actual data relative to the time-shuffled controls. We no longer refer to total durations. l. 314 ff. This is written very vaguely: "elevations were substantial".

We have made the statement more specific as follows: "elevations were substantial (by about an order of magnitude for convergence sizes 7 and 8)." Also, "some underlying mechanisms" - this is both vague and not well motivated.

The sentence was meant to provide a brief interim conclusion to segway to the next paragraphs describing the results pertinent to the potential mechanisms and functions of frequency convergence. As such, the content of the sentence was not meant to be deep, but we agree that "some underlying mechanisms" was unnecessarily vague. We revised the sentence as follows: "Thus, anterior-posterior oscillation-frequency convergences are coordinated rather than coincidental." We hope this is acceptable to the reviewer. l. 449 ff: The method for computing the "linear trend of the phase gradient" needs to be described in detail in the methods section. In the results section, there should be a shorter (yet understandable) description. With the current description, it is not very clear at all what was done here. The linear trend was estimated as the Pearson's r of which two variables? Over which dimension? (e.g. sites? frequencies? times points?) We have augmented the description of how we computed the linear trend of each phase gradient. Given that it is a simple description, we feel that adding it also to the methods section would be redundant. If the reviewer finds the augmented description to be insufficient, please let us know.

Why was the phase gradient weighted by its consistency? Why was PLV taken as a value of consistency? Was the tendency of PLV to report false positives from linear mixing accounted for mathematically? We apologize that our reference to PLV was loose. We now state that PLV reflects the temporal stability of each phase gradient. We agree with the reviewer that PLV reflects source mixing (primarily volume conduction in EEG) in addition to bona fide oscillatory phase locking. To our knowledge, there is no way to computationally remove source-mixing influences from PLV that would be appropriate for our purpose. A common method of extracting the 90{degree sign} component (e.g., iPLV) is effective for detecting the presence of phase locking unconfounded by source mixing, but it would be inappropriate in our case because iPLV, etc., would necessarily underestimate the consistency of phase locking relevant to the phase gradients whose slopes are much lower than 90{degree sign}/site. Moreover, the use of iPLV, etc., would spuriously generate a correlation between phase locking value and phase-gradient slope (iPLV would be necessarily lower for shallower gradients). The only appropriate way to reduce source-mixing influences on PLV in our case is to reduce source mixing via source localization. We used the surface-Laplacian transformation for this reason.

That being said, we looked at the relationship between the absolute slope of phase-gradient and PLV. We found a general positive relationship, statistically significant in all of the minimal-visualstimulation conditions and also in some of the natural-viewing conditions (Extended Data Figure 4-1). The fact that steeper phase gradients tended to have higher PLV's indirectly verifies that larger PLV's in our results reflect greater temporal stability of phase gradients rather than source mixing (which would necessarily reduce phase gradient slopes). We hope that this new analysis alleviates the reviewer's concern. l. 513ff. While this paragraph nicely addresses the question of spurious observations, there are some issues with it:

Volume conduction is only part of the potential sources of linear mixing (source spread being another), although a very large one in EEG. So maybe the section should be titled differently.

We now refer to this as an artifact of source mixing of which volume conduction is a primary contributor in EEG.

At least the equation 1 should be in display, not inline, mode Maybe the equations should be moved to Methods section? We now present equation 1 in a separate line. We prefer to keep the equations where they are as moving them to the methods section would generate redundancy (e.g., having to describe frequency-convergence contingent phase gradients in the methods section).

The results of the control analysis in which slopes were found to be less steep are an important finding and should be shown as extended data. I also recommend that this paragraph be rewritten around the control analysis, so that it read less like a part of the Discussion We now present a plot of the phase gradients comparable to Figure 4D, but computed without applying the surface-Laplacian transformation to EEG data, in Extended Data Figure 4-11. It is clear that the slopes of the phase gradients shown in Extended Data Figure 4-11 are substantially lower than those shown in Figure 4D by about an order of magnitude. We tried revising the section to make it read more like a result section rather than a discussion section. Nevertheless, we believe the mathematical analysis demonstrating that source mixing would only reduce phase-gradient slopes needs to precede the presentation of the supporting result.

We again thank the reviewer for carefully evaluating the revision and giving us very helpful comments and suggestions.
