# Supplementary figures and images for: Characteristics of Spontaneous Anterior–Posterior Oscillation-Frequency Convergences in the Alpha Band
Source: eNeuro. 2025 Mar 25;12(3):ENEURO.0033-24.2025. doi: 10.1523/ENEURO.0033-24.2025 (PMC11949649; doi:10.1523/ENEURO.0033-24.2025)

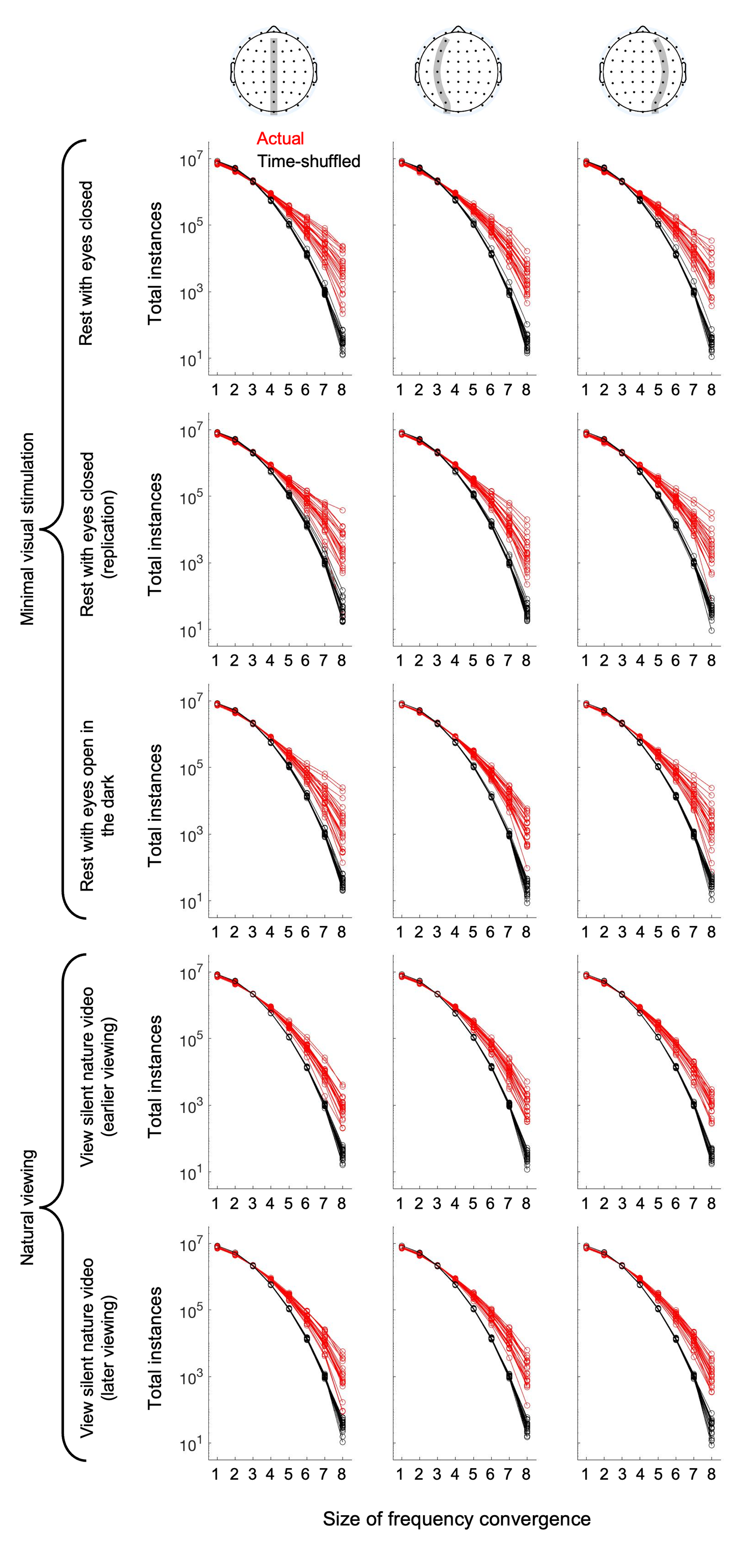

Supplement: Figure 3-1 — The same as Figure 3A, but shows data from all five behavioral conditions. Download Figure 3-1, TIF file. [file eneuro-12-ENEURO.0033-24.2025-s001.tif]

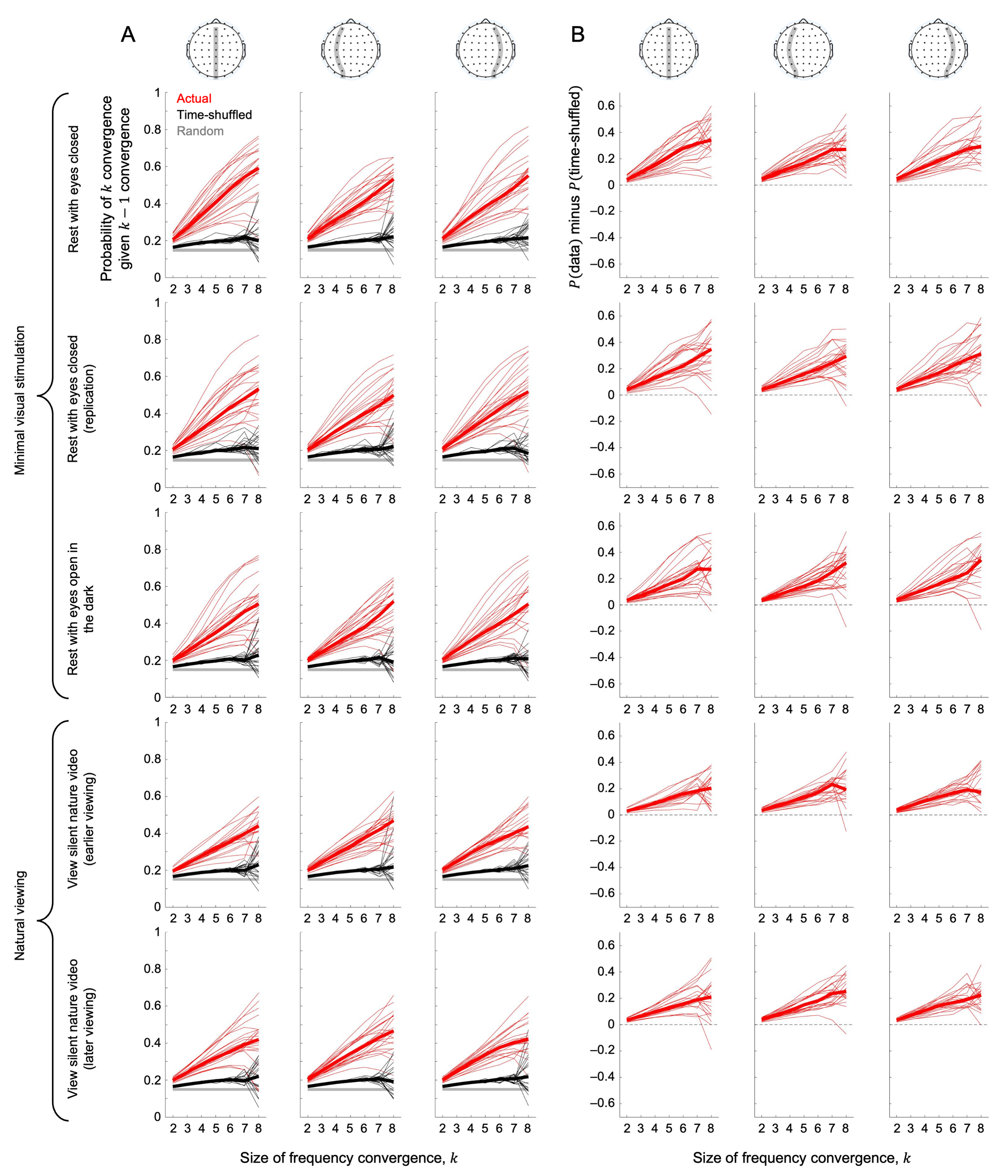

Supplement: Figure 3-2 — The same as Figure 3B and 3C, but shows data from all five behavioral conditions. Download Figure 3-2, TIF file. [file eneuro-12-ENEURO.0033-24.2025-s002.tif]

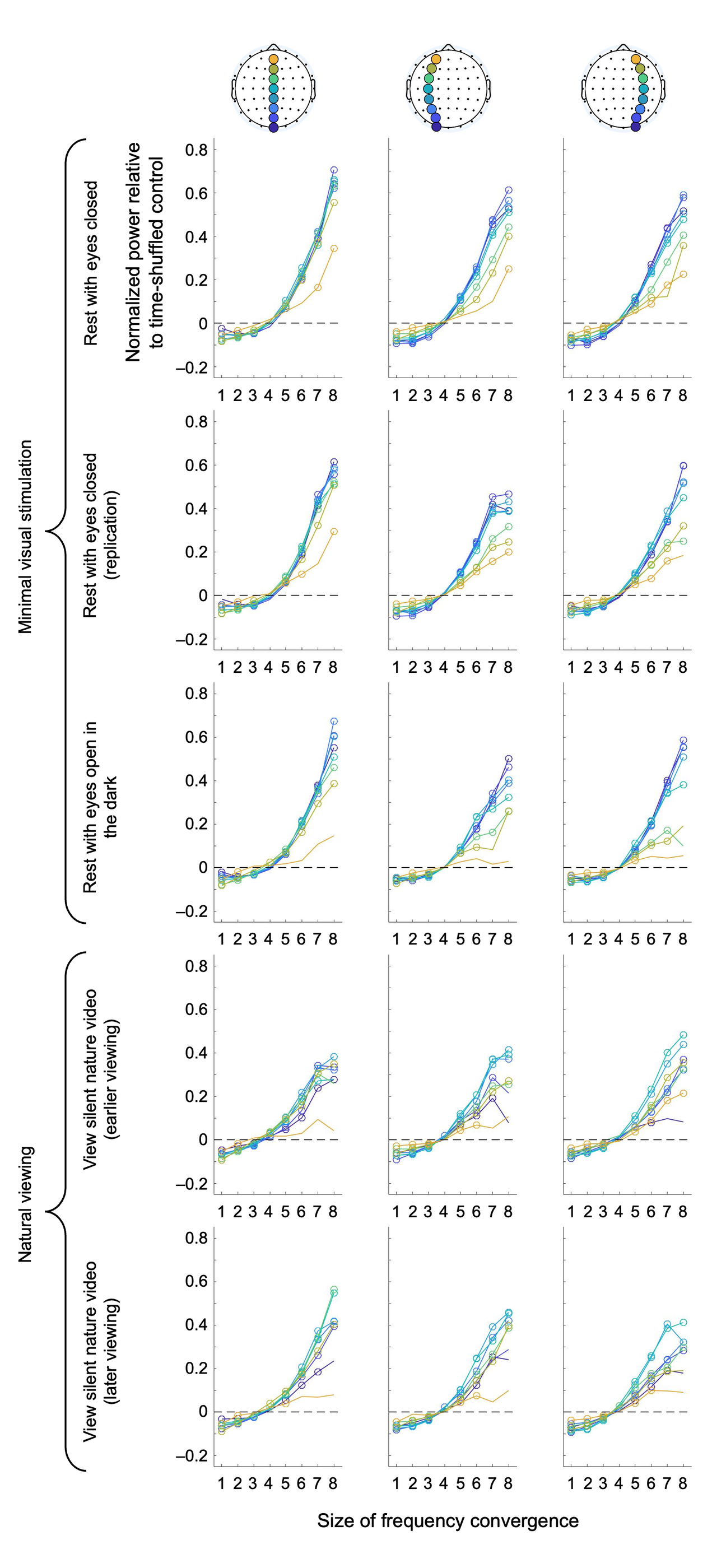

Supplement: Figure 3-3 — The same as Figure 3D, but shows data from all five behavioral conditions. Download Figure 3-3, TIF file. [file eneuro-12-ENEURO.0033-24.2025-s003.tif]

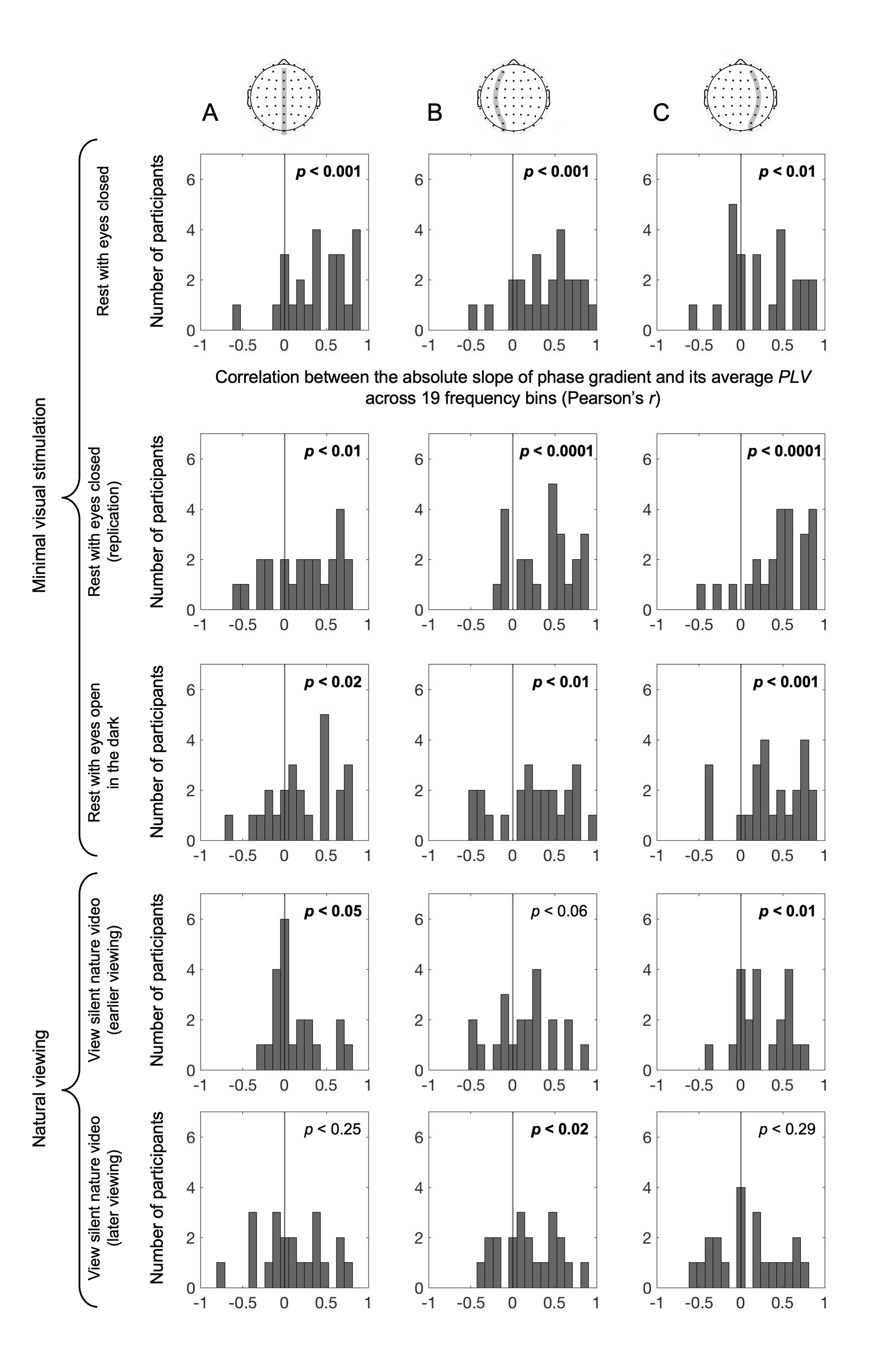

Supplement: Figure 4-1 — Histogram of the correlation (Pearson’s r) between the absolute linear slope of phase gradient (averaged across frequency-convergence sizes) and its average PLV (averaged across all sequential pairwise values within the gradient) across the 19 frequency bins. A. Histograms for the phase gradients along the midline route. B & C. Histograms for the phase gradients along the left-hemisphere and right-hemisphere routes. The rows represent the five behavioral conditions. Note that the average correlation was positive for all anterior-posterior routes and behavioral conditions, and statistically significant in most cases. This result indicates that steeper phase gradients tended to be temporally more stable. Download Figure 4-1, TIF file. [file eneuro-12-ENEURO.0033-24.2025-s004.tif]

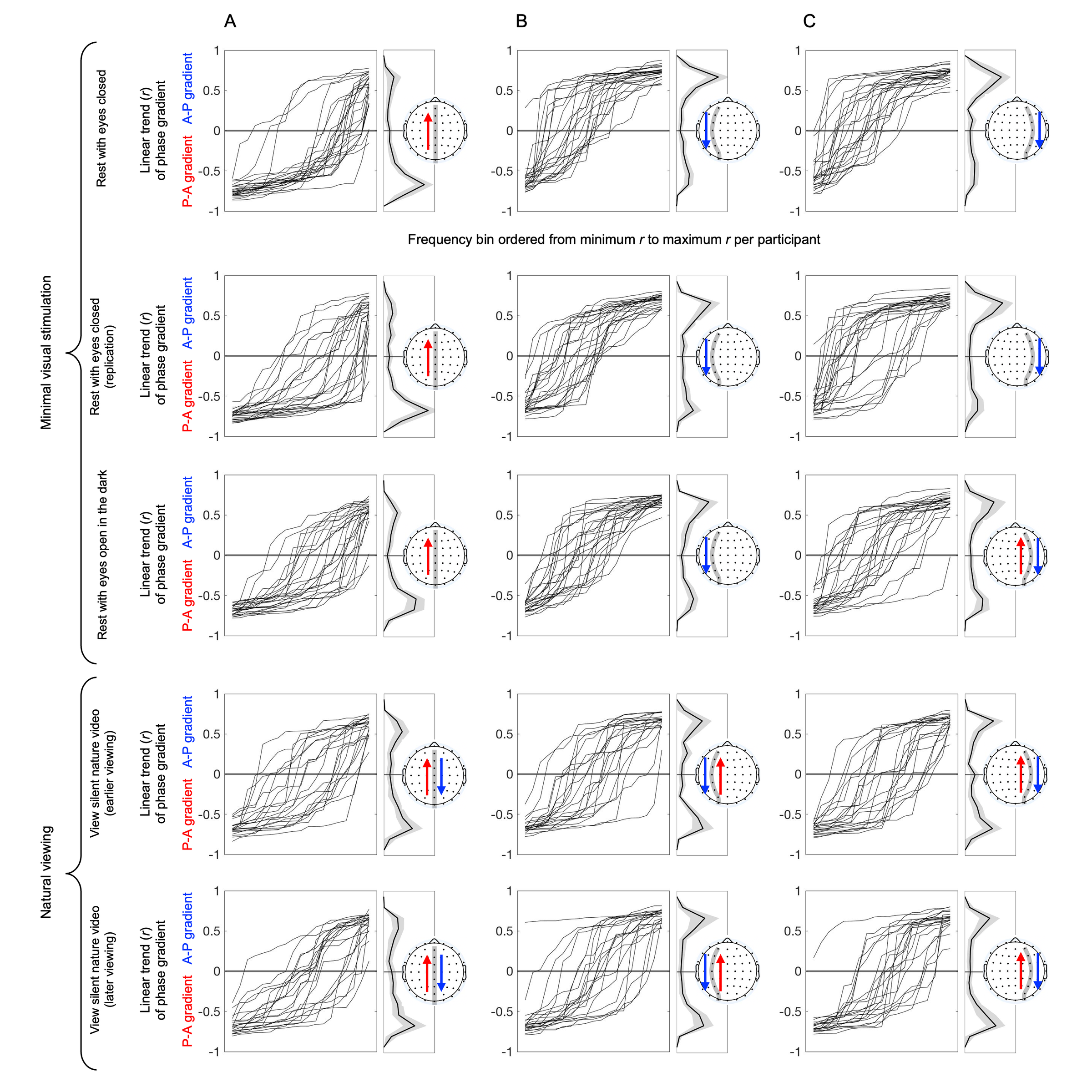

Supplement: Figure 4-2 — Linear trends of the anterior-posterior phase gradients (Pearson’s r weighted by PLV) across the 19 frequency bins (1 Hz wide with 0.5 Hz spacing) ordered from the lowest to highest r value for each participant (a line per participant). A. Phase gradients along the midline route. B & C. Phase gradients along the left- and right-hemisphere routes. Negative values indicate the P-A gradients (illustrated with a single red arrow when they were prevalent) whereas positive values indicate the A-P gradients (illustrated with a single blue arrow when they were prevalent). The histograms (of r values) on the right are the same as those shown in Figure 4A-4C. The rows represent the five behavioral conditions. Note that whether the P-A gradients dominated, A-P gradients dominated, or they formed equally (see the histograms and the red/blue arrows), both types of gradients formed at different alpha frequencies along each of the three anterior-posterior routes for most participants (i.e., most lines traverse high-negative and high-positive r values). Download Figure 4-2, TIF file. [file eneuro-12-ENEURO.0033-24.2025-s005.tif]

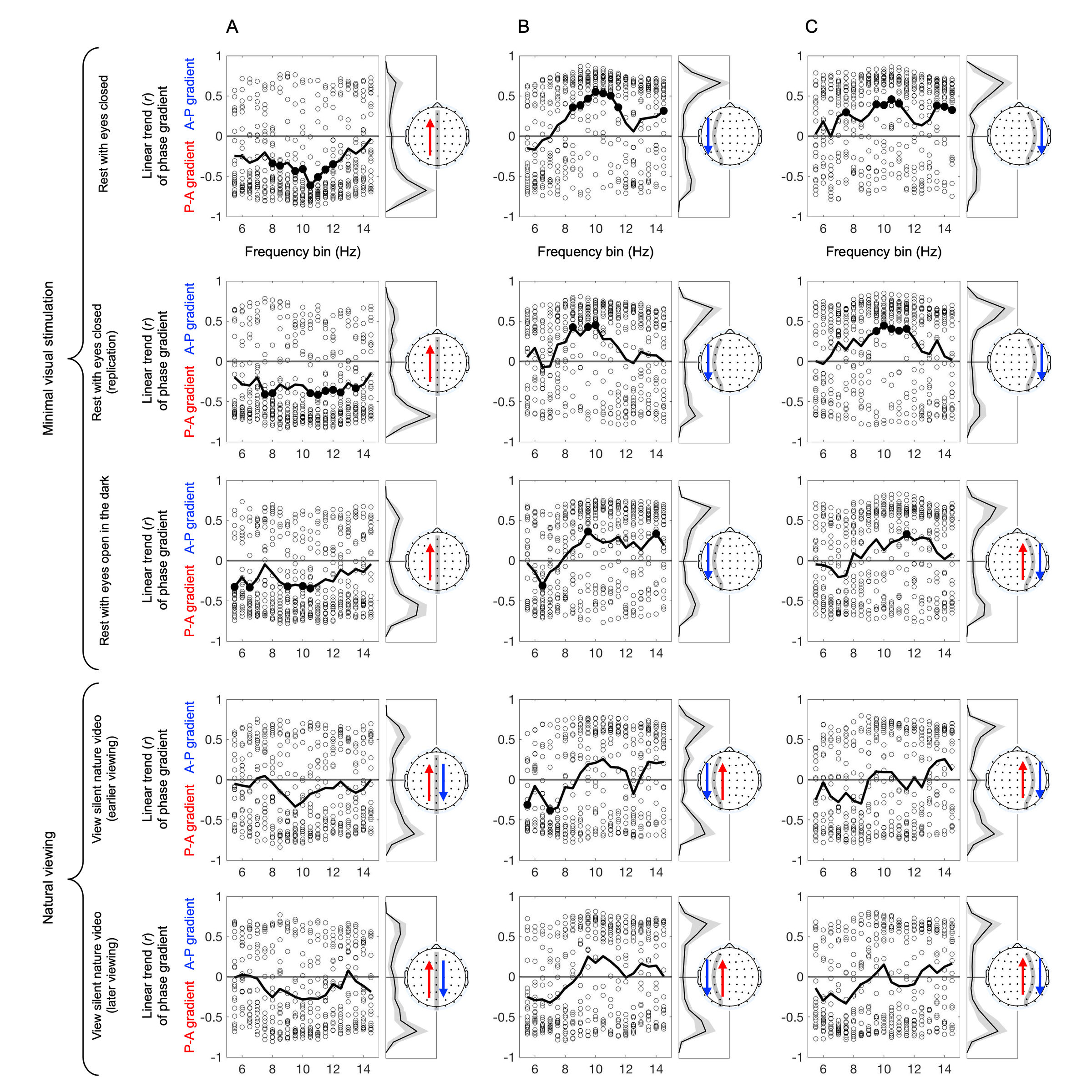

Supplement: Figure 4-3 — Distribution of the linear trend of the anterior-posterior phase gradient (Pearson’s r weighted by PLV) obtained across the 19 frequency bins (1 Hz wide with 0.5 Hz spacing). A. Phase gradients along the midline route. B & C. Phase gradients along the left- and right-hemisphere routes. Negative values indicate the P-A gradients (illustrated with a single red arrow when they were prevalent) whereas positive values indicate the A-P gradients (illustrated with a single blue arrow when they were prevalent). The circles represent data from individual participants with the line representing the mean. The solid circles indicate Bonferroni-corrected statistical significance (α = 0.05, 2-tailed, relative to 0). The histograms (of r values) on the right are the same as those shown in Figure 4A-4C. The rows represent the five behavioral conditions. Note that the prevalent P-A gradients along the midline route (A) and the prevalent A-P gradients along the left- and right-hemisphere routes (B & C) in the eyes-closed conditions (top two rows) are broadly distributed over the alpha range. Download Figure 4-3, TIF file. [file eneuro-12-ENEURO.0033-24.2025-s006.tif]

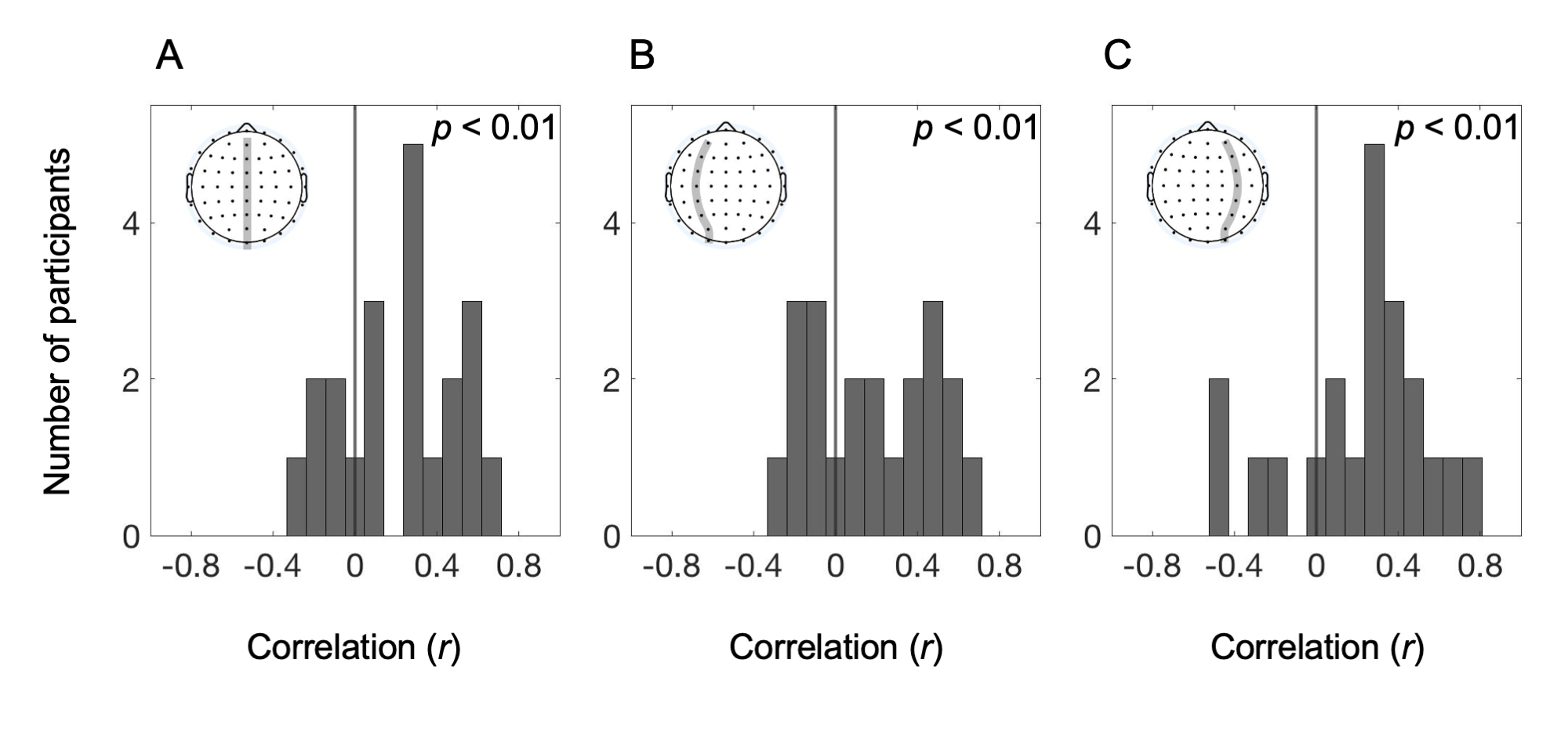

Supplement: Figure 4-4 — Histogram of the within-participant correlation (Pearson’s r) of the pattern of the negative and positive linear trends of the phase gradients across the 19 frequency bins between the earlier and later viewing of a silent nature video. A. The histogram for the midline route. B & C. The histograms for the left- and right-hemisphere routes. All distributions are significantly shifted to the right (the p values reflecting two-tailed t-tests), indicating that the alpha frequencies at which the P-A and A-P gradients formed along each of the three anterior-posterior routes tended to be consistent between the earlier and later viewing of a silent nature video for each participant. Download Figure 4-4, TIF file. [file eneuro-12-ENEURO.0033-24.2025-s007.tif]

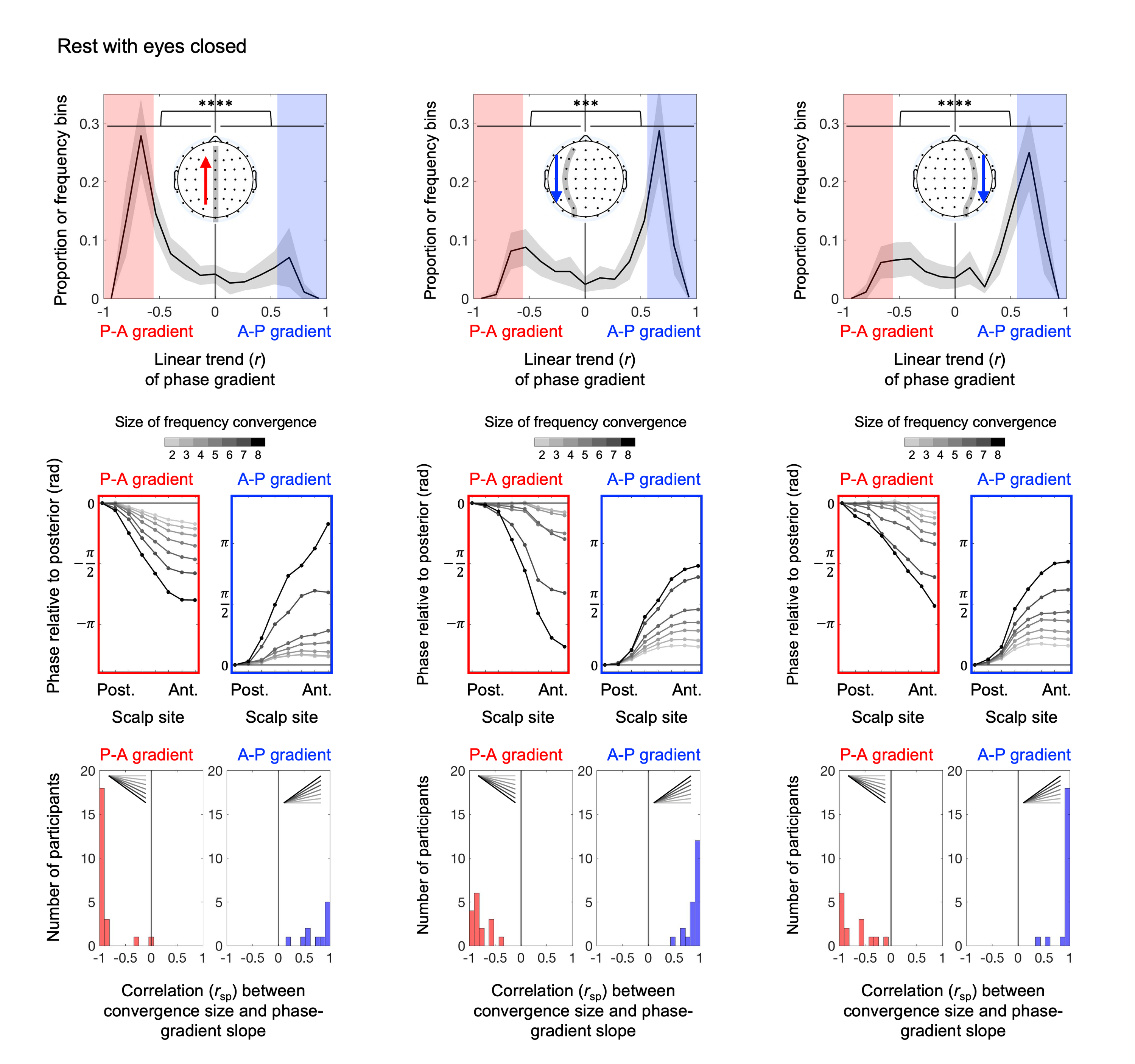

Supplement: Figure 4-5 — The same as Figure 4 but the plots here include only the rest-with-eyes-closed condition. The first row is the same as the first row in Figure 4A-4C. The second row is comparable to Figure 4D and the third row is comparable to Figure 4E. Download Figure 4-5, TIF file. [file eneuro-12-ENEURO.0033-24.2025-s008.tif]

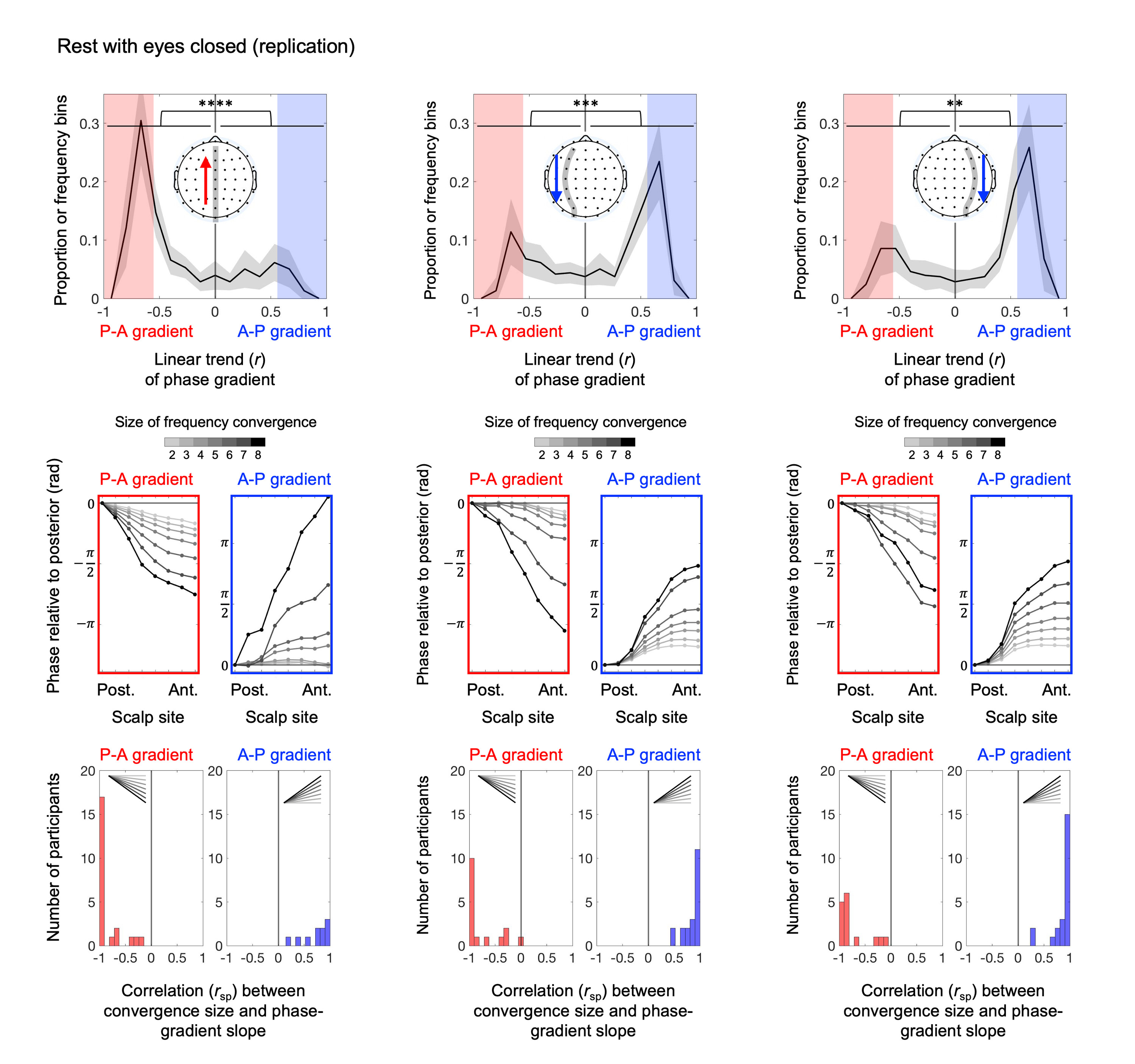

Supplement: Figure 4-6 — The same as Figure 4 but the plots here include only the rest-with-eyes-closed (replication) condition. The first row is the same as the second row in Figure 4A-4C. The second row is comparable to Figure 4D and the third row is comparable to Figure 4E. Download Figure 4-6, TIF file. [file eneuro-12-ENEURO.0033-24.2025-s009.tif]

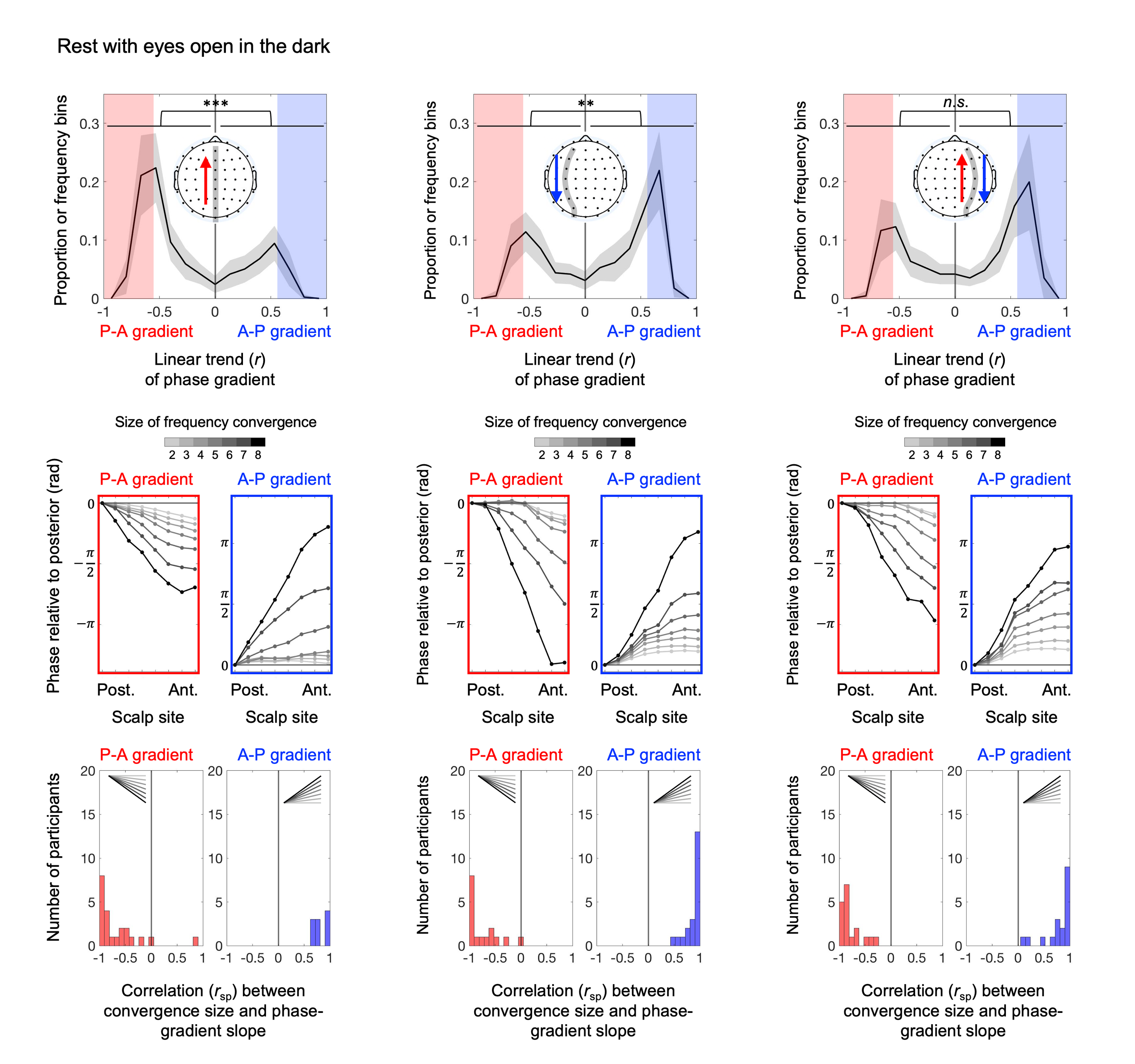

Supplement: Figure 4-7 — The same as Figure 4 but the plots here include only the rest-with-eyes-open-in-the-dark condition. The first row is the same as the third row in Figure 4A-4C. The second row is comparable to Figure 4D and the third row is comparable to Figure 4E. Download Figure 4-7, TIF file. [file eneuro-12-ENEURO.0033-24.2025-s010.tif]

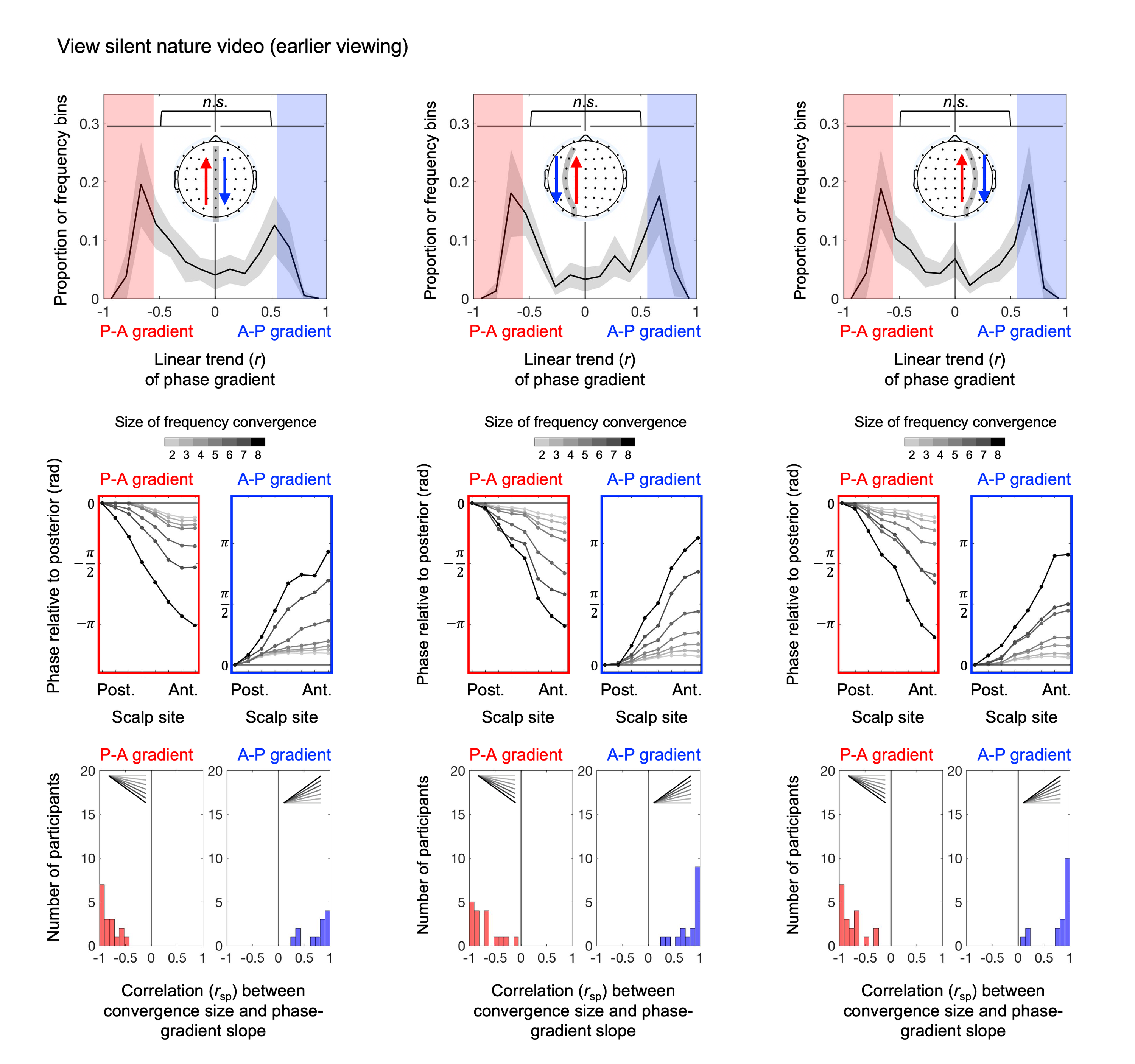

Supplement: Figure 4-8 — The same as Figure 4 but the plots here include only the view-silent-nature-video (earlier viewing) condition. The first row is the same as the fourth row in Figure 4A-4C. The second row is comparable to Figure 4D and the third row is comparable to Figure 4E. Download Figure 4-8, TIF file. [file eneuro-12-ENEURO.0033-24.2025-s011.tif]

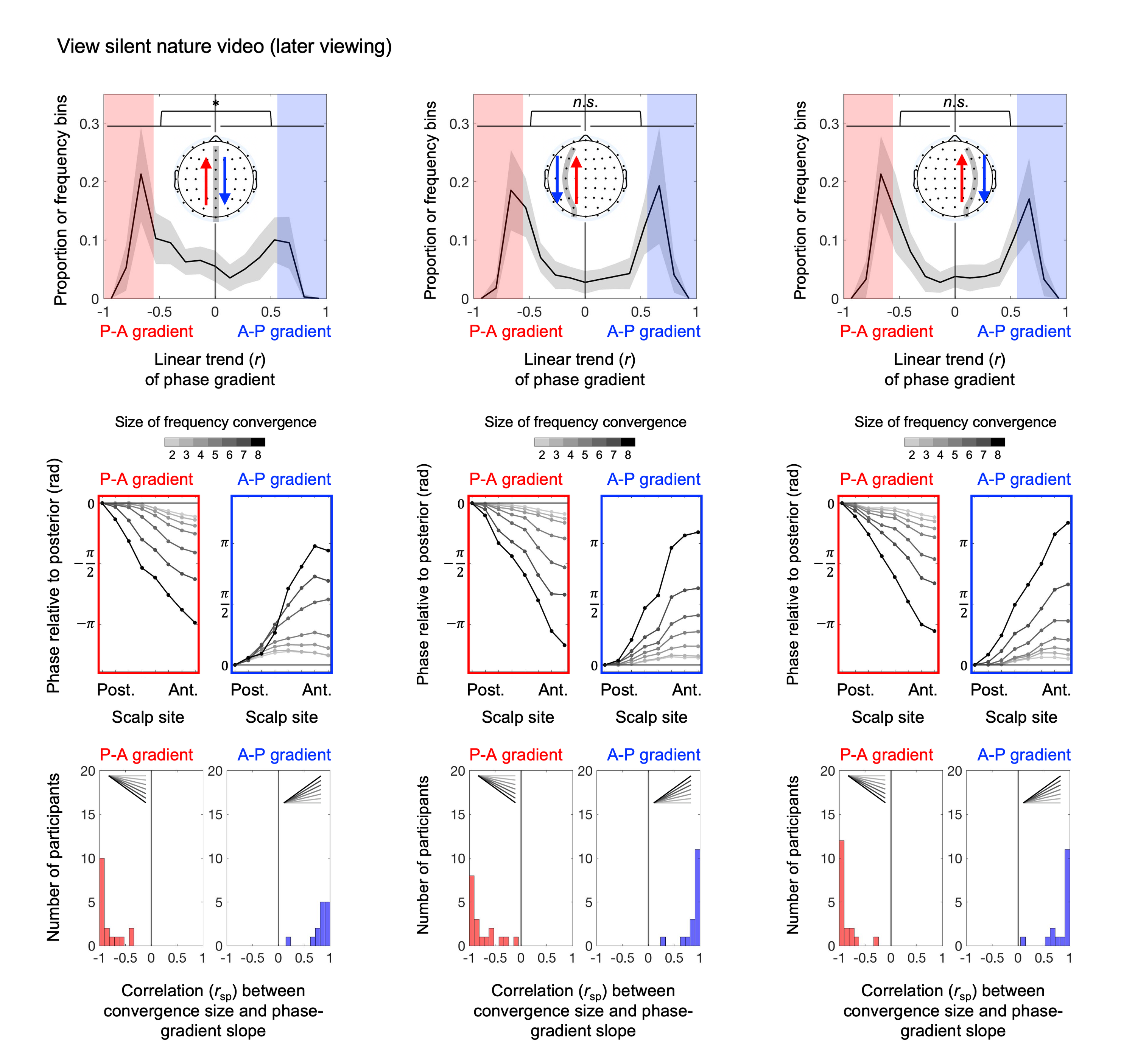

Supplement: Figure 4-9 — The same as Figure 4 but the plots here include only the view-silent-nature-video (later viewing) condition. The first row is the same as the fifth row in Figure 4A-4C. The second row is comparable to Figure 4D and the third row is comparable to Figure 4E. Download Figure 4-9, TIF file. [file eneuro-12-ENEURO.0033-24.2025-s012.tif]

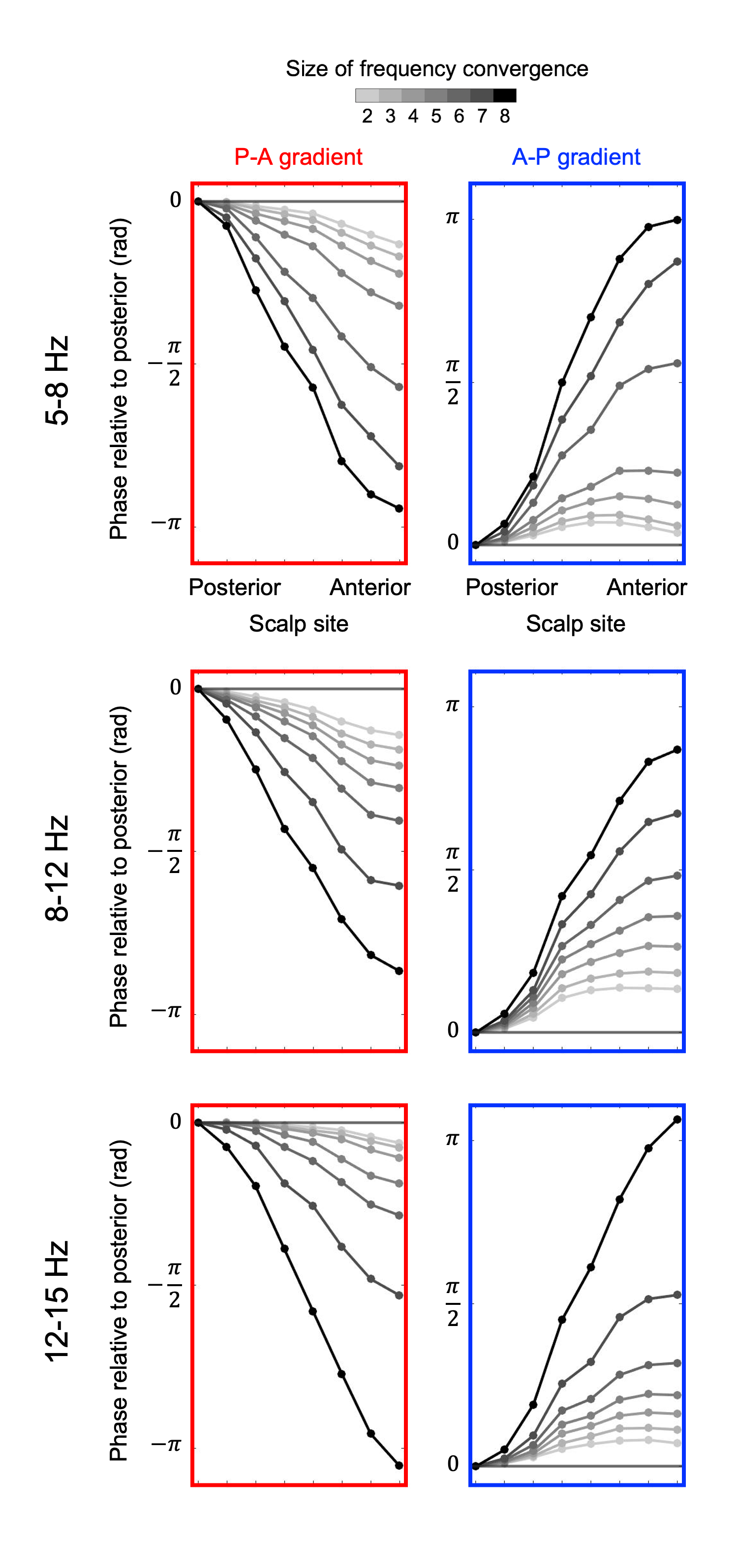

Supplement: Figure 4-10 — The same as Figure 4D, but the P-A and A-P gradients are plotted separately for three alpha subranges: 5-8 Hz, 8-12 Hz, and 12-15 Hz. Note that the gradients are generally similar across the alpha subranges. Download Figure 4-10, TIF file. [file eneuro-12-ENEURO.0033-24.2025-s013.tif]

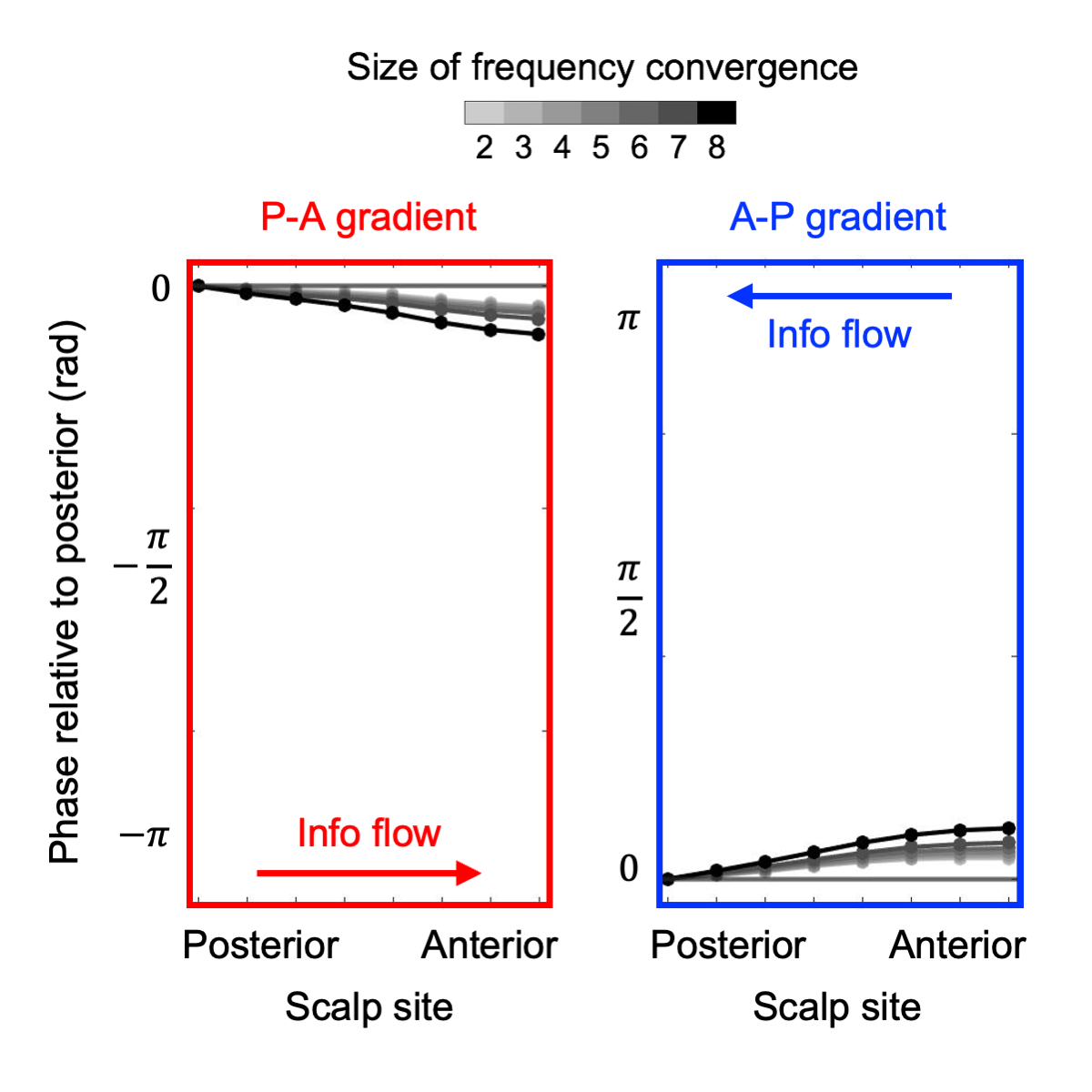

Supplement: Figure 4-11 — The same as Figure 4D, but the analysis was conducted without surface-Laplacian transforming the EEG data. Note that the phase-gradient slopes are decreased relative to Figure 4D by a factor of ∼10. Download Figure 4-11, TIF file. [file eneuro-12-ENEURO.0033-24.2025-s014.tif]
